# Evaluation of environmental factors and microbial community structure in an important drinking-water reservoir across seasons

**DOI:** 10.3389/fmicb.2023.1091818

**Published:** 2023-02-14

**Authors:** Jie Feng, Letian Zhou, Xiaochao Zhao, Jianyi Chen, Zhi Li, Yongfeng Liu, Lei Ou, Zixin Xie, Miao Wang, Xue Yin, Xin Zhang, Yan Li, Mingjie Luo, Lidong Zeng, Qin Yan, Linshen Xie, Lei Sun

**Affiliations:** ^1^State Environmental Protection Key Laboratory of Drinking Water Source Management and Technology, Shenzhen Academy of Environmental Sciences, Shenzhen, China; ^2^GeneMind Biosciences Company Limited, Shenzhen, China

**Keywords:** metagoenomics, trophic level index (TLI), cylindrospermopsins (CYNs), subtropical drinking water source, multidrug resistance genes

## Abstract

The composition of microbial communities varies in water and sediments, and changes in environmental factors have major effects on microbiomes. Here, we characterized variations in microbial communities and physicochemical factors at two sites in a large subtropical drinking water reservoir in southern China. The microbiomes of all sites, including the diversity and abundance of microbial species, were determined *via* metagenomics, and the relationships between microbiomes and physicochemical factors were determined *via* redundancy analysis. The dominant species in sediment and water samples differed; *Dinobryon* sp. LO226KS and *Dinobryon divergens* were dominant in sediment samples, whereas Candidatus *Fonsibacter ubiquis* and *Microcystis elaben*s were dominant in water. The diversity was also significantly different in microbial alpha diversity between water and sediment habitats (*p* < 0.01). The trophic level index (TLI) was the major factor affecting the microbial community in water samples; *Mycolicibacterium litorale* and *Mycolicibacterium phlei* were significantly positively related to TLI. Furthermore, we also studied the distribution of algal toxin-encoding genes and antibiotic-resistant genes (ARGs) in the reservoir. It found that water samples contained more phycotoxin genes, with the cylindrospermopsin gene cluster most abundant. We found three genera highly related to cylindrospermopsin and explored a new cyanobacteria *Aphanocapsa montana* that may produce cylindrospermopsin based on the correlation through network analysis. The multidrug resistance gene was the most abundant ARG, while the relationship between ARGs and bacteria in sediment samples was more complicated than in water. The results of this study enhance our understanding of the effects of environmental factors on microbiomes. In conclusion, research on the properties, including profiles of algal toxin-encoding genes and ARGs, and microbial communities can aid water quality monitoring and conservation.

## 1. Introduction

Reservoirs are essential for managing imbalances in the supply and demand for water resources (Wu et al., 2021). In recent decades, the release of pollutants from river tributaries (e.g., sewage discharge) has stimulated the accumulation of nutrients and algal growth, leading to eutrophication and reductions in water quality (Wurtsbaugh et al., 2019). Blooms of algae, such as cyanobacteria, in reservoirs occur frequently, and this poses a threat to the safety of drinking water sources and induces major economic losses (Paerl, 2018). The size, frequency, and duration of harmful algal blooms are increasing, which has been driven in part by human activities, such as habitat degradation and global climate change (Sakamoto et al., 2021). Harmful algal blooms pose considerable threats to water quality and drinking water security because they are difficult to predict; gauging the deleterious effects of the toxins produced by algae during such blooms is also a grand challenge (Park et al., 2021). Harmful algal blooms affect the composition of microbial communities and various aspects of the reservoir environment (Park et al., 2021). Several challenges are associated with studying the ecotoxicology of reservoirs because they are natural sinks for contaminants derived from atmospheric fallout (Wiesner-Friedman et al., 2021). Reservoirs can also provide a favorable environment for the reproduction of microorganisms serving as a bioreactor. Thus, it is important to characterize the composition of microbial communities in reservoirs.

Yantian Reservoir was built in 1976 (22°40′3′′N, 114°9′48′′E; altitude 83 m). It lies in a medium-sized subtropical basin at the junction of the Shenzhen and Dongguan regions of the South China Basin. The reservoir draws water from the Shima River Basin, a tributary of the Dongjiang River in the tropical and subtropical regions of Guangdong. The reservoir has an area of 25.6 km^2^ and a storage capacity of 8.99 million m^3^; it provides potable water for residents of the Pearl River Delta (Wang et al., 2019). The reservoir provides drinking water for 1.3 million people and supplies 350,000 m^3^ of water per day. It also plays an important role in flood control. Nutrient concentrations in adjacent inflow rivers have increased because of the rapid development of township corporations over the last few decades (Wang et al., 2019). The reservoir’s waters are vulnerable to eutrophication and algal blooms, and the aquatic ecosystem is deteriorating into simplified food networks with inferior water quality (Wang et al., 2019). Given this situation, a 10-km^2^ restoration project was initiated in 2015 to remove excess nutrients and inhibit algal blooms in the eutrophic bay of the reservoir, which involved stocking fish and increasing areal coverage of submerged vegetation (Wang et al., 2019). Planting submerged hydrophytes and stocking herbivorous, planktivorous, and molluscivorous fish enhanced water quality by stabilizing plankton micro-ecosystems and reducing the content of nutrients. Although ecological restoration measures of this pilot project have been completed, the water quality and composition of microbial communities in this reservoir have not yet been evaluated. Given that Yantian Reservoir is the most important water supply for the urban population in the Pearl River Delta and several ecological restoration projects have been carried out, the water quality and structure of microbial communities of the reservoir require evaluation.

Surface water is the primary source of potable water, and the quality of surface water has considerable human health implications (Long and Luo, 2020). Algal toxins, antibiotic-resistant genes (ARGs), and 2-methylisoborneol pose major health risks (Van Dolah, 2000). The abundance of antibiotic-resistant bacteria (ARB) has increased in recent years because of the widespread use of antibiotics. The ARGs contained in ARB have become recognized environmental pollutants. The uptake of ARGs by pathogens and the development of antibiotic resistance pose threats to human health. Given that the long-term storage of water in reservoirs increases the retention and accumulation of ARGs (Dang et al., 2020), studies of the distribution and diffusion of ARGs in reservoirs are required to control ARG pollution and mitigate health risks.

Diverse types of organisms are essential for maintaining the stability of freshwater ecosystems. Microbial communities are affected by various environmental factors, and the relationships between microbial communities and environmental factors are indicators of the function and structure of aquatic ecosystems (Mucha et al., 2004; Boucher et al., 2006; Pound et al., 2021). However, changes in microbial communities in water sources are complicated by upstream water input and reservoir function (Jiang et al., 2021). For example, microbes can affect the Earth’s energy fluxes and diverse biogeochemical cycling pathways in lakes and reservoirs (Sjöstedt et al., 2012; Penn et al., 2014; Savvichev et al., 2018; Bobkov et al., 2021). Next-generation sequencing (NGS), bioinformatics, and functional genomic studies have revealed a high diversity of microorganisms in lakes and reservoirs (Qin et al., 2016). Previous studies have also revealed high similarity in the predominant microorganisms in various water ecosystems; however, the abundances of these microorganisms likely vary with water quality (Jiang et al., 2021). The abundances of dominant species have also been shown to vary with depth and among sampling locations (Chien et al., 2009). Here, we used metagenomic sequencing to assess the variation in the composition of microbial communities in Yantian Reservoir. We also characterized the distribution of algal toxins and ARGs in Yantian Reservoir to provide information that could aid water quality monitoring efforts.

## 2. Materials and methods

### 2.1. Collection of water and sediment samples

Water samples were collected weekly from October 2020 to January 2021 in a subtropical drinking water reservoir in southern China (Supplementary Figure S1). Samples were collected from two sites: the center of the reservoir (KZ) and the water intake (QSK). Water samples were collected using 3-L brown glass bottles at a depth of 0.5 m. They were then taken to the laboratory and maintained at 4°C. All samples were filtered *via* a 0.45-μm polycarbonate membrane (Collins, Shanghai, China) prior to chemical analysis. Sediments were sampled following the methods described in a previous study (He et al., 2015), and the sampling frequency was twice a month from October 2020 to December 2020.

### 2.2. Water physicochemical properties

Measurements were taken on the following parameters: water temperature, pH, dissolved oxygen (DO), transparency, permanganate index (PI), ammonia nitrogen (NH3.N), total nitrogen (TN), total phosphorus (TP), and chlorophyll *a* (Elser et al., 2007). Measurements of water quality parameters were taken following national standard specifications (Water quality—Determination of pH—Electrode method HJ1147-2020, Water quality—Determination of dissolved oxygen—Electrochemical probe method HJ 506-2009, Seville disc method, Water quality-Determination of permanganate index GB/T 11892-1989, Water quality—Determination of ammonia nitrogen—Salicylic acid spectrophotometry HJ 536-2009, Water quality-Determination of total nitrogen-Alkaline potassium persulfate digestion UV spectrophotometric method HJ 636-2012, Water quality-Determination of total phosphorus-Ammonium molybdate spectrophotometric method GB/T 11893-1989, and Water quality—Determination of chlorophyll *a*—Spectrophotometric method SL88-2012). The tropic level index (TLI) of water samples was calculated using TN, TP, permanganate index, transparency, and chlorophyll *a* indicators and equations from a previous study (Ding et al., 2021). There were five trophic levels in the lake: hypereutrophic (TLI > 70), middle eutrophic (60 < TLI ≤ 70), mesotrophic (30 ≤ TLI ≤ 50), and oligotrophic (TLI < 30).

### 2.3. DNA extraction and library sequencing

DNA was extracted from membrane filters and frozen sediment samples by the FastDNA® SPIN Kit for Soil (MP Biomedicals, United States) according to the manufacturer’s protocols. The NanoDrop One instrument (Thermo Fisher Scientific, United States) was used to determine DNA concentrations and purity. Shotgun metagenomic sequencing of the DNA library was carried out concurrently. In brief, 30 micrograms of DNA was processed to build a library, which was then sequenced using the GeneLab M sequencing platform.

### 2.4. Bioinformatics analysis

A total of 26 samples were sequenced (Supplementary Table S1) and annotated to a database of freshwater algae in China^1^ withmore than 200 freshwater algal genomes to characterize the composition of freshwater algae in Yantian Reservoir. This database was used to identify algal taxa in our samples. Reads that could not be mapped to the freshwater algae genome were aligned to the reference database Kraken2. The Chao1, ACE, Shannon, and Simpson indices (Edwards et al., 2001) were computed using the vegan package in R. Principal component analysis (PCA) was carried out using the FactoMineR package in R. Pearson correlation coefficients were calculated to evaluate correlations among variables. The pheatmap package in R was used to build a correlation heatmap. The vegan, FactoMineR, and pheatmap packages were all implemented in R. Differential abundance analysis was performed using the DESeq2 Bioconductor package (v.1.36.0) in R with default parameters. Significant species were identified using the following criteria: *p* < 0.05 and fold change >2 or <1/2. All samples were integrated into the global network according to the count data. Species that were present in all samples (total number greater than 50,000) were subjected to correlation analyses. Correlations were considered significant using the following criteria: Spearman correlation coefficients (*R* > 0.6) and (*p* < 0.01). Networks were visualized using Cytoscape software. Sequences of the 10 most dominant species with log_2_ (fold change) > 1 and *p* < 0.05 were obtained from NCBI. Clean reads were aligned to these genes using Bowtie2 v2.4.5 to confirm their presence in our samples [at least 18 and 34 reads were aligned in sediment samples (*n* = 9) and water samples (*n* = 17), respectively]. Kyoto Encyclopedia of Genes and Genomes (KEGG) analysis of these genes was conducted by aligning them against the KEGG database using DIAMOND software. A diagram of the results of the KEGG pathway analysis was made using the ggplot2 package in R.

### 2.5. ARG-like and algal toxin gene abundance calculations

The abundance of ARGs was calculated by ARGs-OAP v2.0 (Yin et al., 2018) based on the sequencing reads. For algal toxins, 16 common algal toxin gene clusters were selected (Supplementary Table S2) to calculate the abundance. The heatmap was drawn by standardizing the read counts of the algal toxin gene cluster as log10 (value*1000000 + 0.001).

### 2.6. Correlation networks

A transboundary symbiosis network consisting of the algal toxin genes or ARG and genus was established to assess species’ coexistence in different habitats and regions. We focused on the ones in all samples to reduce rare absolute abundance (SAV) in the data set. Robust correlations with Spearman’s correlation coefficients (*p*) >0.8 or <−0.8 and *p* < 0.001 were used to construct networks, which have been extensively used in the literature and are comparable across studies (Delgado-Baquerizo et al., 2020). The Gephi interactive platform^2^ was used to visualize the network.

## 3. Results

### 3.1. Physicochemical properties of samples

The physicochemical characteristics of the water samples were determined (Table 1). Temperature and TLI ranged from 17.4 to 26.5°C and from 48.56 to 58.63 in QSK and from 16.8 to 24.8°C and from 48.12 to 51.77 in KZ, respectively. The TLI and temperature were higher in most of the QSK samples than in the KZ samples (e.g., on November 3 and 23). The pH ranged from 7.89 to 8.74 in QSK and from 7.79 to 9.45 in KZ. The difference in pH between QSK and KZ samples was small. Over the study period, TLI and chlorophyll *a* increased (>20), and the Secchi disc depth gradually decreased (<0.5 m). Generally, differences in the physicochemical properties of samples among months were not pronounced. Because Shenzhen features a subtropical to tropical oceanic climate, temperature differences between October 20 and January 21 are not substantial. High temperatures are more conducive to the reproduction of algae and bacteria, which poses major risks to drinking water safety. The TLI values of QSK samples were higher than those of KZ samples at the same sampling times, indicating that algae and bacteria were more abundant in QSK than in KZ.

**Table 1:** Value of environmental variables measured in the samples of water.

	Sample name	Temperature of water (°C)	pH	Dissolved oxygen (mg/L)	Permanganate index (mg/L)	Ammonia nitrogen (mg/L)	Total nitrogen (mg/L)	Total phosphorus (mg/L)	Secchi disc depth (M)	Chlorophyl a (μg/L)	TLI	Eutropher
KZ	W_KZ201103	24.80	7.97	7.85	1.40	0.02	1.07	0.06	0.51	31	49.32	Mesotropher
	W_KZ201116	24.30	8.01	7.99	2.60	0.03	1.04	0.07	0.47	12	50.27	Light eutropher
	W_KZ201123	22.50	8.33	8.14	2.10	0.03	1.11	0.05	0.41	28	51.34	Light eutropher
	W_KZ201130	22.90	8.05	8.93	1.30	0.05	1.09	0.07	0.48	22	48.71	Mesotropher
	W_KZ201207	20.10	8.30	9.48	1.80	0.04	1.24	0.07	0.45	24	51.17	Light eutropher
	W_KZ201214	20.00	8.59	9.90	2.20	0.03	1.24	0.04	0.49	32	50.97	Light eutropher
	W_KZ201229	20.80	9.45	11.76	2.30	0.04	1.16	0.04	0.56	14	48.12	Mesotropher
	W_KZ210104	16.80	8.74	11.61	2.60	0.03	1.20	0.05	0.52	28	51.77	light eutropher
QSK	W_QSK201020	26.50	8.74	9.71	3.30	0.02	0.80	0.14	0.35	45	57.63	Light eutropher
	W_QSK201103	25.70	8.00	7.99	1.90	0.01	0.96	0.09	0.45	36	52.60	Light eutropher
	W_QSK201116	24.30	7.89	7.90	3.30	0.02	0.90	0.08	0.47	20	52.88	light eutropher
	W_QSK201123	22.50	8.52	8.23	2.30	0.01	0.94	0.09	0.43	26	52.69	Light eutropher
	W_QSK201130	23.00	8.01	8.82	1.30	0.05	1.11	0.10	0.45	13	48.56	Mesotropher
	W_QSK201207	19.80	8.52	9.58	1.90	0.02	1.18	0.10	0.43	28	52.98	light eutropher
	W_QSK201229	19.10	8.54	9.78	2.20	0.03	1.12	0.05	0.53	14	48.68	Mesotropher
	W_QSK210104	17.40	8.62	10.75	2.70	0.04	1.23	0.05	0.55	16	50.21	Light eutropher
	T-test (*p* value)	0.19	0.70	0.37	0.36	0.18	0.01	0.01	0.26	0.85	0.17	

### 3.2. Microbial community composition and diversity

**Figure 1:**
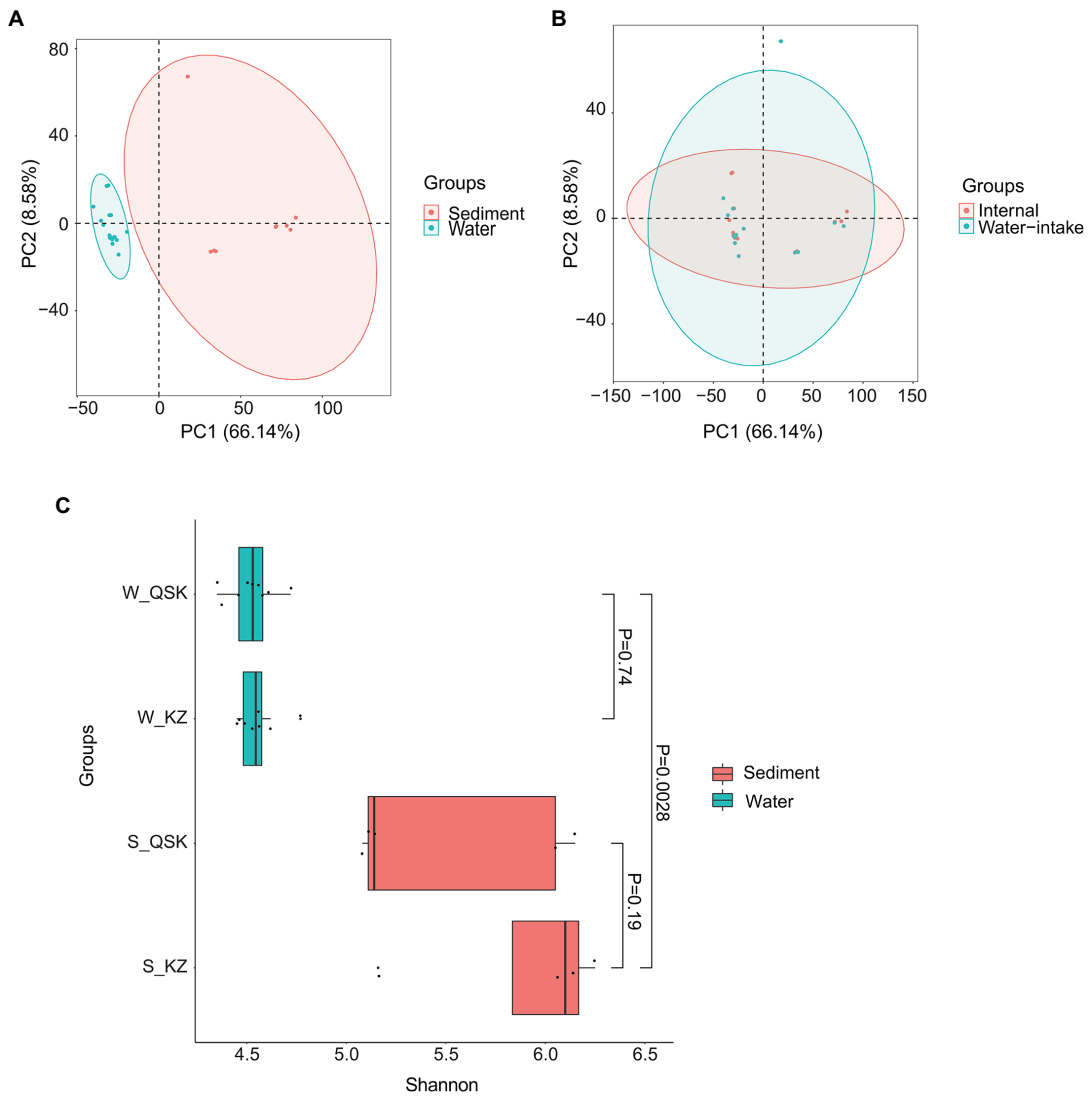
PCA and alpha diversity of sediment samples and water samples. **(A)** PCA of all samples, **(B)** PCA of water samples; **(C)** Shannon index of water and sediment samples.

The microbial communities in sediment and water samples differed (Figure 1A; MRPP: Chance corrected within-group agreement A: 0.4801; observed delta 0.1902 and expected delta 0.3659; significance of delta: 0.001). No differences were observed in samples collected at different times and sampling sites (Figure 1B). Shannon alpha diversity values did not differ among sediment samples from different sites nor water samples from different sites (Figure 1C). The alpha diversity of the sediment samples was significantly higher than that of the water samples (*p* < 0.01, Wilcoxon rank-sum test). No significant differences were observed in the alpha diversity of KZ and QSK in sediment or water samples (Table 2). Pearson correlation coefficients revealed weak correlations between sediment and water samples in alpha diversity; however, intra-group correlations were high (Supplementary Figure S2). Sample origin (water vs. sediment) had a greater effect on microbial alpha diversity than sampling time or the sampling site. This might stem from the stable climate of Shenzhen, which experiences little variation in water temperatures (21.91 ± 2.89; Table 1). Seasonal shifts did not significantly decrease or increase water temperature; seasonal variation thus did not affect microbial alpha diversity. Algae can influence water quality; we thus analyzed algal alpha diversity. The alpha diversity of algae was greater in sediment samples than in water samples (Supplementary Table S3). The diversity of algae was greater in the QSK than in the KZ. We found that TLI was higher in QSK than in KZ. In conclusion, obvious differences in microbial communities were observed between sediment and water samples. Although sampling location and time did not substantially affect algal alpha diversity, the algal alpha diversity of QSK was higher than that of KZ.

**Table 2:** Relative abundance and diversity of all species community.

Sample name	Observed_species	Chao1	ACE	Shannon	Simpson
W_QSK201020	4,304	4,594	4,452	4.35	0.07
W_QSK201207	4,439	4,708	4,610	4.61	0.05
W_QSK201116	4,351	4,617	4,489	4.46	0.07
W_QSK201123	4,386	4,671	4,547	4.53	0.06
W_QSK201103	4,333	4,586	4,466	4.58	0.05
W_KZ210104	4,304	4,552	4,441	4.77	0.03
W_KZ201123	4,384	4,677	4,533	4.62	0.05
W_KZ201207	4,418	4,835	4,603	4.56	0.06
W_QSK201214	4,366	4,644	4,526	4.72	0.05
W_KZ201214	4,392	4,665	4,543	4.46	0.06
W_KZ201116	4,360	4,635	4,508	4.45	0.07
W_QSK210104	4,351	4,678	4,504	4.56	0.04
W_KZ201103	4,340	4,562	4,472	4.56	0.05
W_KZ201130	4,336	4,655	4,472	4.53	0.06
W_QSK201229	4,338	4,576	4,480	4.37	0.07
W_QSK201130	4,355	4,630	4,505	4.50	0.06
W_KZ201229	4,311	4,568	4,447	4.49	0.07
S_KZ201130	4,597	5,241	4,917	6.25	0.01
S_QSK201020	4,267	4,522	4,415	5.11	0.06
S_KZ201229	4,562	5,025	4,829	6.06	0.01
S_QSK201130	4,352	4,643	4,507	5.08	0.04
S_KZ201214	4,450	4,875	4,660	5.16	0.03
S_QSK201103	4,396	4,733	4,587	5.14	0.04
S_QSK201229	4,526	5,034	4,794	6.15	0.01
S_QSK201214	4,501	4,874	4,705	6.05	0.01
S_KZ201103	4,545	5,117	4,837	6.14	0.01

Shannon, Simpson: diversity indice. ACE, Chao: community richness indice.

### 3.3. Variation in microbial abundance

**Figure 2:**
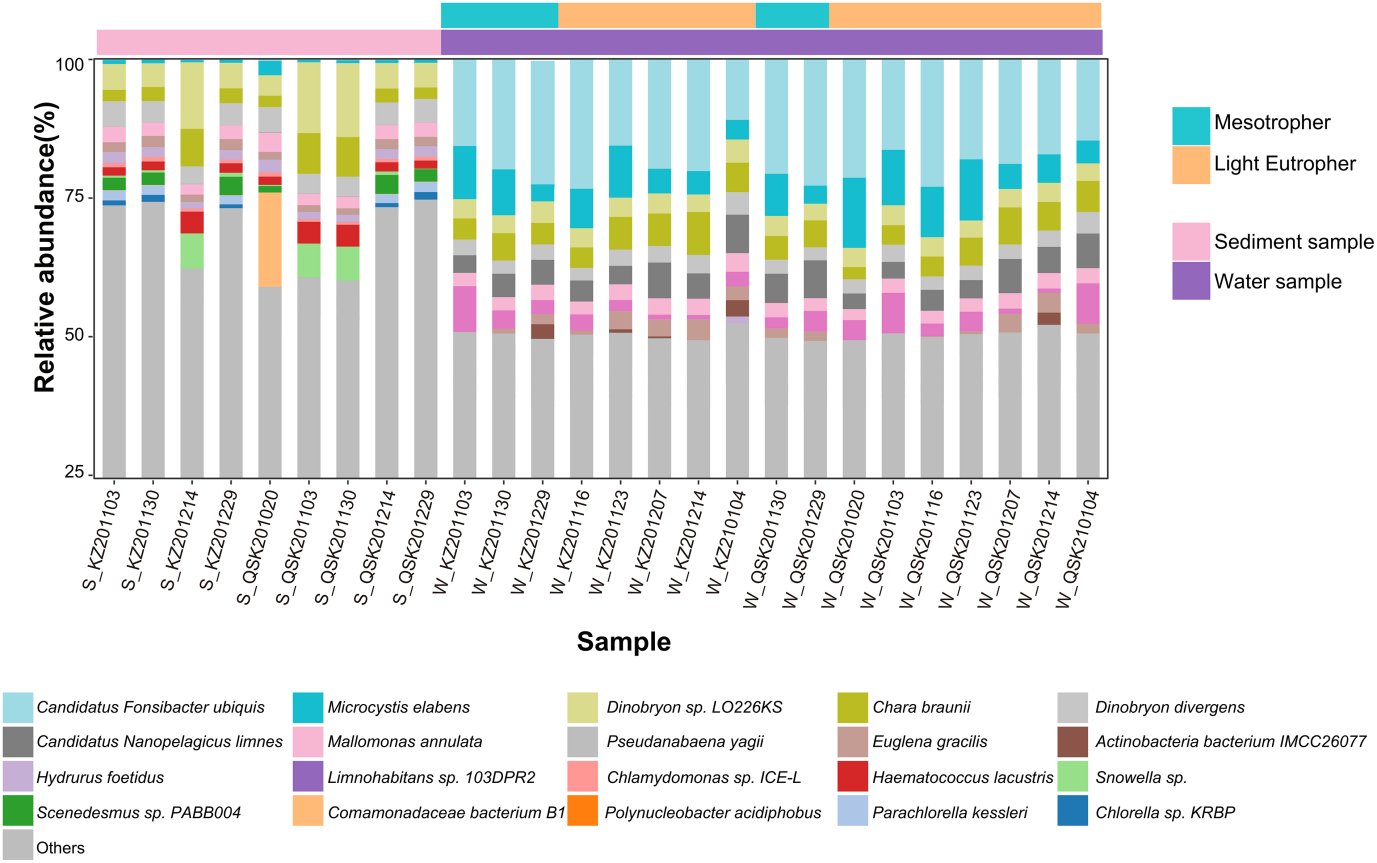
Histogram of relative abundances at the species level. The 20 most abundant species are shown, and “Other” indicates species with relative abundances less than 0.6% across all samples.

The relative abundances of microbes were calculated at the species level (Figure 2). Overall, the composition of microbial communities in sediment and water samples differed significantly. The dominant species in the sediment samples were *Dinobryon* sp_LO226KS, *Dinobryon divergens*, *Chara braunii*, and *Mallomonas annulata*. *Candidatus Fonsibacter ubiquis*, *Pseudanabaena yagii*, *Chara braunii*, and *Microcystis elabens* were dominant species in water samples. Based on reads, more bacteria were detected in water samples. For example, *Candidatus Fonsibacter ubiquis* and *Candidatus Nanopelagicus limne* were only identified in water samples. Little variation was observed in the composition of the microbial community under different trophic levels; variation in TLI among samples was also low (mesotrophic 48.68 ± 0.43 and light eutrophic 52.23 ± 2.05). A total of 14 of the 20 most abundant species were algae. The abundances of the 20 most abundant algae were determined. The average relative abundance of algae was higher in water samples than in sediment samples (DESeq2, *p* < 0.05; Supplementary Figure S3). *Chara braunii* and *Euglena gracilis* were dominant in water samples, whereas *Haematococcus lacustris* and *Snowella* sp. were dominant in sediment samples. These findings reinforce that the composition of microbial communities differed in sediment and water samples.

Benthic cyanobacteria can be a nuisance because of their potent toxins, as well as tastes and odours they can impart to the water. The dominant taxa of benthic cyanobacteria have changed little over time (Supplementary Figure S4), suggesting that seasonal change had less impact on these taxa in Yantian Reservoir. Additionally, all sediment samples exhibited a similar dominating genera *Oscillatoria, Cyanobium*, *Leptolyngbya*, and *Microcystis.*

**Figure 3:**
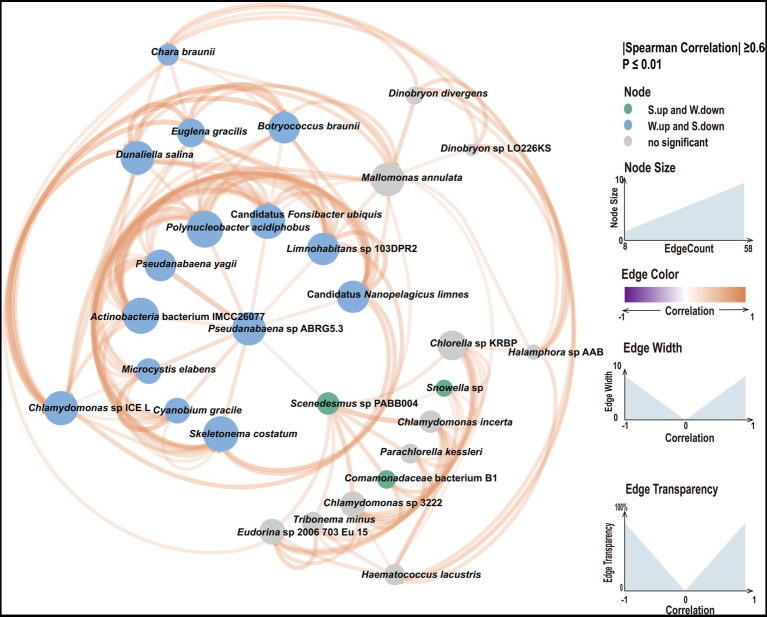
Network analysis of dominant species with significant differences among samples. The thickness and thinness of the lines indicate strong and weak correlations, respectively. Orange lines indicate positive correlations, and gray lines indicate negative correlations. Blue circles indicate taxa with higher abundances in water samples, and green circles indicate taxa with higher abundances in sediment samples.

Differences in the abundances of species between water and sediment were determined. Significant differences in the abundance of species were detected for 2,411 species (Supplementary Table S4). The average abundance of most species was greater in water samples than in sediment samples (61.47%). We focused on species with high abundance and with significant differences in abundance between water and sediment samples. *Snowella* sp., *Comamonadaceae bacterium* B1, and *Scenedesmus* sp. PABB004 best met these criteria in sediment samples; *Candidatus Fonsibacter ubiquis*, *Candidatus Nanopelagicus limnes,* and *Limnohabitans* sp. 103DPR2 best met these criteria in water samples. We conducted KEGG analysis to characterize functional differences between water and sediment samples (Supplementary Figure S5). Differences in KEGG pathways were observed between water and sediment. The main KEGG pathways in the water samples were related to photosynthesis and pigment synthesis, whereas, in sediments, the main KEGG pathways were related to flagella formation. *Candidatus Fonsibacter ubiquis* and *Candidatus Nanopelagicus limnes* were dominant in water samples, and *Snowella* sp. was dominant in sediment samples. These findings suggest that sediment and water samples might mutually affect each other. Therefore, we constructed a network based on abundance differences and correlation coefficients (Figure 3). In the network analysis of dominant species, we found that *Pseudanabaena* sp. ABRG5.3 occupies a key position in the entire network, *Pseudanabaena* sp. ABRG5.3 is positively correlated with numerous species in water samples and negatively correlated with *Scenedesmus* sp. PABB004, and *Scenedesmus* sp. PABB004 is a key specie in the sediment samples and positively correlated with species in sediment samples, such as *Snowella* sp. These findings indicate that the abundances of dominant species in water and sediment samples mutually influence each other.

### 3.4. Effect of water physicochemical factors on microbial communities

**Figure 4:**
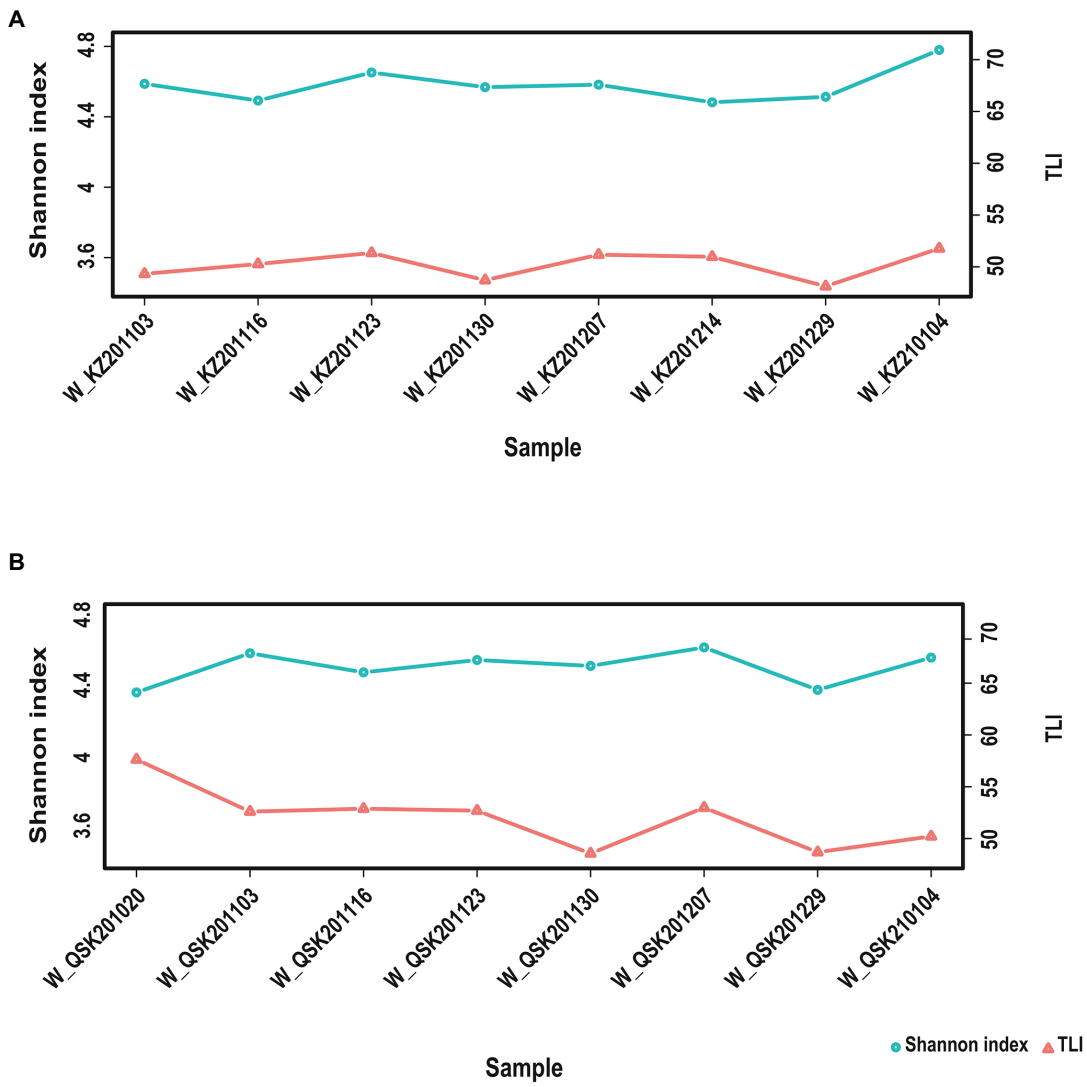
Relationships between TLI and the Shannon index. **(A)** KZ samples, **(B)** QSK samples.

**Figure 5:**
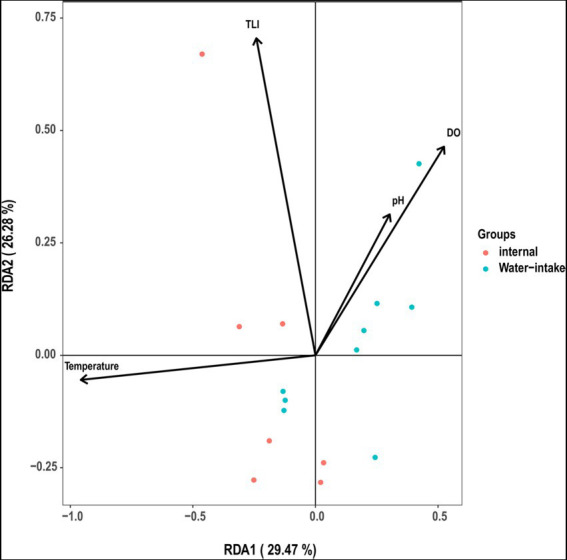
Redundancy analysis ordination diagram of all communities and environmental variables. Circles correspond to samples.

**Figure 6:**
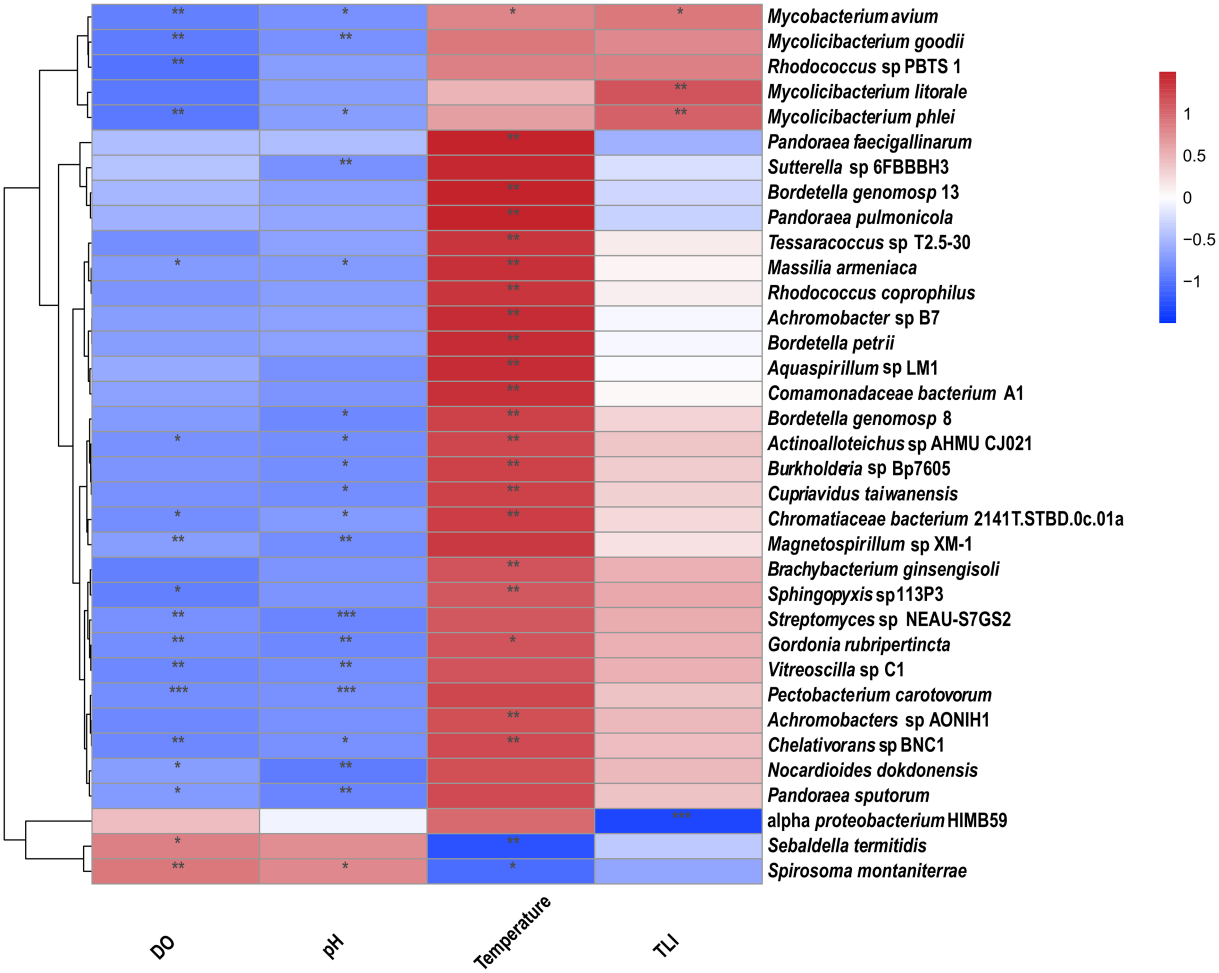
Correlation heatmap for microbes and environmental factors. The magnitude of the *R*-values is shown in the color gradient. *p ≤ 0.05, **p ≤ 0.01, and ***p ≤ 0.001.

TLI is widely used to evaluate the magnitude of eutrophication of rivers, reservoirs, and lakes in China (Ding et al., 2021). We found that changes in the TLI in KZ were similar to changes in the Shannon index; exceptions to this general pattern were only observed at two time points (2020-11-03 and 2020-12-29; Figure 4). This suggests that TLI might be a key environmental factor affecting species diversity. The relative abundance of *Microcystis elabens* gradually decreased over time, consistent with the changing trend of water temperature. RDA was performed to identify the key environmental factors affecting microbial diversity. The amount of variation in the data explained by RDA1 and RDA2 was similar (Figure 5). The long length of the arrow for TLI indicates that it was most strongly correlated with microbial communities, followed by temperature and DO. Temperature and TLI were nearly perpendicular to each other, meaning they were not correlated. The angle between DO and TLI was less than π/2, which suggests that they might be weakly positively correlated. The relationship between water physicochemical factors and species was explored. The number of species that were strongly positively correlated with temperature and TLI was greater than the number of species that were negatively correlated with temperature and TLI (Figure 6). The abundances of *Mycolicibacterium litorale* and *Mycolicibacterium phlei* were significantly positively correlated (*p* < 0.01), and alpha-proteobacterium HIMB59 was significantly negatively correlated with TLI (*p* < 0.001). We also analyzed correlations of these species with other environmental factors to provide information for follow-up studies (Supplementary Figure S6). Species were significantly positively or negatively correlated with TP and PI. By contrast, only species significantly negatively correlated with ammonia-N and TN could be found. This might indicate that ammonia-N negatively affects biodiversity.

### 3.5. Algal toxin gene clusters

**Figure 7:**
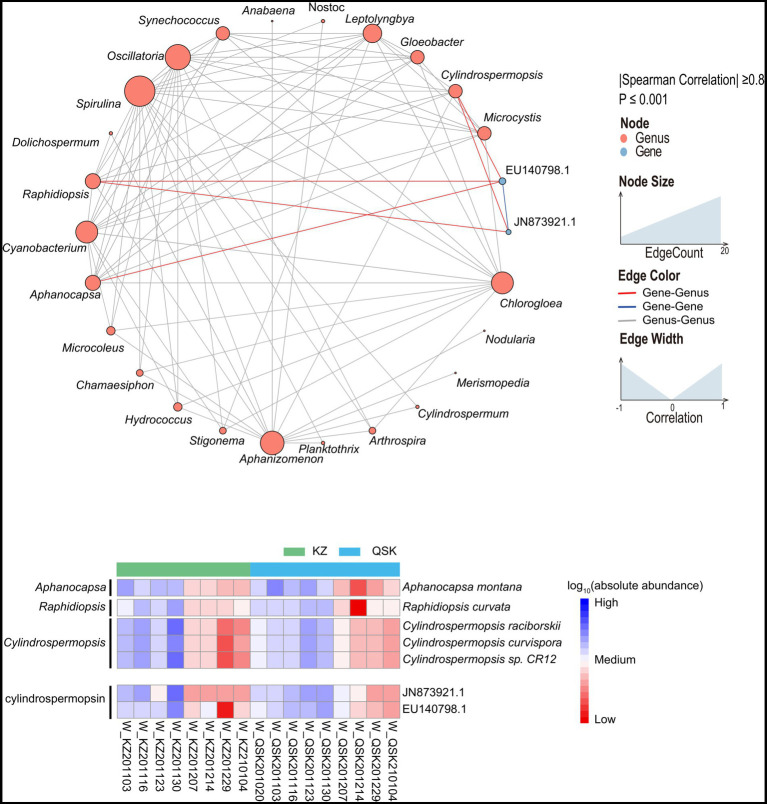
Cross-genus co-occurrence network of phycotoxin gene clusters in water samples. Network analysis coloring based on taxonomic genus preference. Associations indicated a significant (*p* < 0.001), scored as positive (spearman’s *p* > 0.8). The size of each node is proportional to the SAV degree; the thickness of the connection between two nodes is proportional to the value of the Spearman correlation coefficient. Heatmap of the relative abundances of algal toxin gene clusters in all samples.

The relative abundances of algal toxin gene clusters were identified from reservoir samples. Three classes of five algal toxin gene clusters were detected, and the abundances of these gene clusters were higher in water samples than in sediment samples (Supplementary Table S2 and Supplementary Figure S7). The abundances of algal toxin gene clusters were highest in November 2020. The relative abundance of cylindrospermopsin (CYN) gene clusters was the highest among all algal toxin gene clusters and comprised 98% of mapping reads. Two CYN gene clusters *EU140798.1* and *JN873921.1* were found to be prevalent among all the water samples and they were strongly correlated with three genera, including *Cylindrospermopsis*, *Raphidiopsis* and *Aphanocapsa* (Figure 7 up). The absolute abundances of these three genera and algal toxins were calculated to analyze the likely source species of algal toxins (Figure 7 down). It revealed the changes in the abundance of *Raphidiopsis curvata, Cylindrospermopsis raciborskii, Cylindrospermopsis curvispora*, and *Cylindrospermopsis* sp. CR12 were consistent with the changes in the abundance of the two CYN gene clusters. The sample W_KZ201214 had the highest abundance of gene cluster. Few studies have indicated that *Aphanocapsa montana* contains the algal toxin gene cluster *EU140798.1* while a sequence similar to the algal toxin gene cluster *EU140798.1* was found to be present in the genome of *A. montana* (Supplementary Table S5). The TLI of these samples was also high, except the sample W_KZ201130, suggesting that a high TLI might promote algal growth and lead to a rise in the abundance of algal toxin genes in water.

### 3.6. Abundances of ARGs

**Figure 8:**
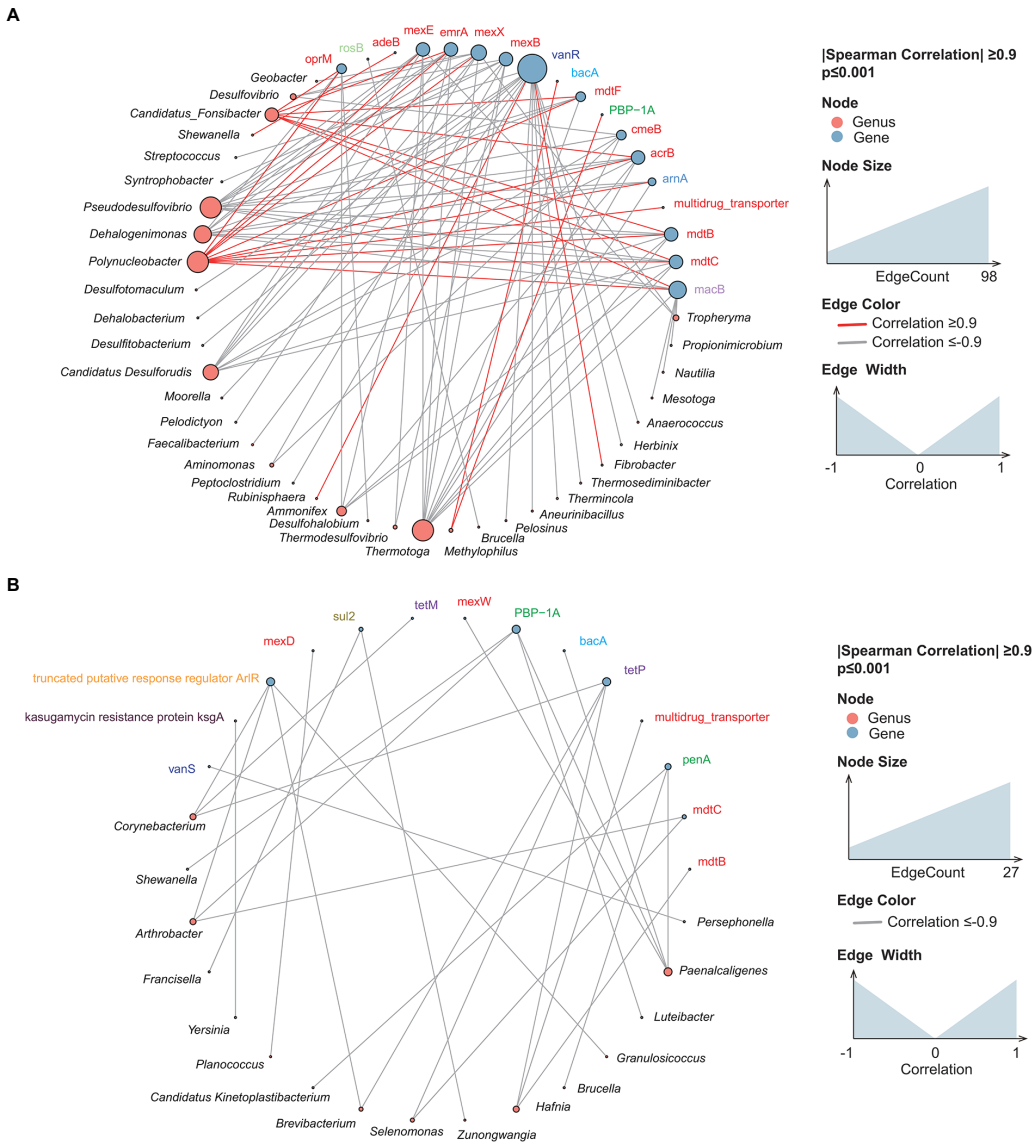
Cross-genus co-occurrence network of ARGs in samples. Network analysis coloring based on taxonomic genus preference. Associations indicated a significant (*p* < 0.001), scored as positive (spearman’s *p* > 0.9 or *p* < −0.9). The size of each node is proportional to the SAV degree; the thickness of the connection between two nodes is proportional to the value of the Spearman correlation coefficient. **(A)** Sediment samples; **(B)** water samples.

In recent years, a growing number of reservoir-related studies have examined ARGs (Li et al., 2015; Dang et al., 2020). In the present investigation, the distribution of ARGs in sediment and water samples was similar (Supplementary Figure S8). The three most abundant types of ARG genes—multidrug resistance (MDR), macrolide-lincosamide-streptogramin, and beta-lactam ARGs—accounted for over 70.5% of all ARGs. More than 95% of all the ARGs were derived from Proteobacteria, Actinobacteria, and Firmicutes. The relative abundance of ARGs carried by Firmicutes was 4.11% lower in water samples than in sediment samples; the relative abundance of ARGs from Actinobacteria was 20.54% higher in water samples than in sediment samples. Multidrug (−0.36%) and beta-lactam (+0.22%) ARGs were the most common Actinobacteria-derived ARGs. The multidrug-resistant bacteria were mainly in the family Enterobacteriaceae (relative abundance over 53%). To explore the potential hosts of ARGs, we investigated the correlation between ARGs and bacterial genera. The results indicated that the network relationship in sediment samples was far more complex than in water (Figure 8). Under the same screening criteria, there were more interspecies connections in the sediment samples, and more species and ARG types were present. *Polynucleobacter* in sediment samples was positively correlated with multiple multidrug resistance genes such as *mdtB* and *mdtC*. Likewise, *Paenalcaligenes* in water samples was positively associated with multiple drug-resistance genes.

## 4. Discussion

Clarifying associations between microbial communities and water quality is essential for enhancing the understanding of reservoir characteristics. In this study, the structure of microbial communities was explored through continuous monitoring of the water and sediment of drinking water reservoirs at two locations in southern China over a 4-month period. We found that the dominant species in sediment samples and water samples differed, which is consistent with the results of previous studies (Yang et al., 2012; El Najjar et al., 2020; Nwosu et al., 2021). We also found that the composition of microbial communities in the reservoir was not significantly affected by seasonal factors, which might stem from the lack of variation in the water temperature of Yantian Reservoir among seasons. On the one hand, a relatively stable water temperature can facilitate the study of the effects of other environmental factors, such as TLI, TP, and TN on the composition of microbial communities in reservoirs. On the other hand, the growth of many microorganisms is enhanced when reservoir water temperatures are maintained above 18°C, increasing the health risks posed by algal toxins and ARGs. MDR genes were the most abundant ARGs in water and sediment samples. Algal toxin genes were more abundant in water samples than in sediment samples.

A four-year ecological restoration project was initiated at Yantian Reservoir in October 2014, and completed in December 2017. During this period, a 10,000-m^2^ ecological restoration area was constructed to reduce nutrient loading and mitigate the consequences of eutrophication and to provide food and habitat for fish and mollusks (Wang et al., 2019). Fish were harvested to maintain system stability. Despite project completion, changes in water quality and the composition of microbial communities following the partial restoration of Yantian Reservoir have not been investigated. This study details the results of data collected over several years on the microbial communities in this reservoir, as well as various environmental factors. No pre-ecological restoration project samples were obtained; thus, no comparisons before and after ecological restoration are possible. However, data were obtained on the water quality and composition of microbial communities in Yantian Reservoir 4 years after ecological restoration measures had been completed. These data are essential for evaluating the effects of the ecological restoration project on water quality.

Water quality is a major environmental priority for modern society (Alcamo, 2019). Regular water quality monitoring is essential for ensuring the safety of drinking water, an adequate food supply, and the health of human populations. Basic field kits with multiple sensors, fluorescence, spectrophotometric, and microscopic-based mobile technologies have been used to characterize the composition of microbial communities and estimate water quality. These can be used to detect microbes (bacterial concentrations) and chemicals (Grossi et al., 2013), fecal coliforms, pathogenic parasites, heavy metals, and pesticides (Acharya et al., 2020). However, these methods only provide preliminary insights into the composition of microbial communities in water. NGS permits more comprehensive characterizations of the water microbiome. Metagenomic approaches permit real-time quantification of microbial hazards in sewage (Aarestrup and Woolhouse, 2020). Therefore, the use of metagenomic techniques for monitoring water quality merits further study. The excessive growth of cyanobacteria because of eutrophication is becoming increasingly widespread, leading to deterioration in water quality (Huisman et al., 2018). The health threats posed by algal toxins have raised concerns among health organizations and water authorities (Van Dolah, 2000). Microcystin, a well-known algal toxin, might be associated with developing irritant reactions, hepatic diseases, and the progression of tumors (Tamele and Vasconcelos, 2020). CYN can impair the liver, heart, kidney, and thymus (Terao et al., 1994) by inhibiting protein synthesis (Froscio et al., 2008) and glutathione metabolism (Runnegar et al., 1995). CYN is also a potential carcinogen that inhibits pyrimidine nucleotide synthesis (Reisner et al., 2004) and induces DNA strand breakage (Bazin et al., 2012). Although we detected algal toxin gene clusters, such as CYN and microcystin, in Yantian Reservoir in this study, the association between algal toxins and species could not be accurately quantified because we did not attempt to detect chemical substances in the samples. In future studies, we plan to monitor the relationships between phycotoxins and algal species.

In this study, species from genera, *Cyanobium*, *Leptolyngbya*, *Microcystis*, and *Oscillatoria* predominated in all sediment samples. Species of these four genera are reported to produce algal toxins (Frazão et al., 2010) and benthic cyanobacteria can cause taste and odour concerns in the water (Frazão et al., 2010; Casero et al., 2019; Entfellner et al., 2022). Thus, monitoring benthic cyanobacteria should be added to monitoring programs advocating drinking water safety (Izaguirre et al., 2007; Gaget et al., 2017, 2020).

Although the health risks posed by ARGs are a worldwide concern, research in this field is lacking (Hu et al., 2021). Increases in the abundance of ARGs in reservoirs affect the safety of drinking water and the efficacy of clinical antibiotics therapy (Ghai et al., 2014; Nnadozie and Odume, 2019; Dang et al., 2020). Previous studies of reservoirs in eastern China have revealed that high concentrations of ARGs can be detected in the water after being transported over long distances (Hu et al., 2018; Hu and Lin, 2018). We also identified ARGs in Yantian Reservoir, and the most abundant ARGs were MDR genes, which is consistent with the results of a previous study of Danjiangkou Reservoir (Dang et al., 2020). Firmicutes, which are dominant intestinal microorganisms, were the second largest source of ARGs of all microbial phyla in Yantian Reservoir; their presence in large numbers indicates that the water quality of Yantian Reservoir poses serious human health risks. The problem of drug resistance in MDR pathogens has attracted clinical attention because these pathogens result in increased morbidity and mortality, especially in immunocompromised populations (Catalano et al., 2022). Enterobacteriaceae was the primary source of MDR genes in a previous study (Dang et al., 2020). The occurrence and spread of multidrug-resistant Enterobacteriaceae pose public health threats because available treatment options are limited, and the development of new antimicrobial agents is historically slow (Cerceo et al., 2016; Chen et al., 2019). The widespread occurrence of MDR genes in reservoirs poses major threats to human health.

## 5. Conclusion

Metagenomic approaches were used to characterize the composition of microbial communities and environmental factors in water and sediment samples in Yantian Reservoir across seasons at two different locations. Seasonal changes and sampling location did not have major effects on the composition of microorganisms in Yantian Reservoir; however, sample origin (water vs. sediment) had a substantial effect on the composition of microorganisms. The distribution of algal toxins and ARGs was analyzed as were the source species of algal toxins. The distribution of algal toxins and ARGs in Yantian Reservoir was also studied, and then based on correlation analysis, the potential sources species of algal toxins and ARGs were identified. This study revealed a new potential source of cyanotoxins through the colonial cyanophyte *Aphanocapsa montana*. The study also found that TLI is a key factor affecting the composition of microbes in Yantian Reservoir and identified the species most strongly correlated with TLI.

## Data availability statement

The datasets presented in this study can be found in online repositories. The names of the repository/repositories and accession number(s) can be found in the article/Supplementary material.

## Author contributions

LS and LX conceived and designed the research, reviewed and revised the manuscript. JF and XZhao wrote the manuscript. YLiu and QY reviewed and revised the manuscript. JC, ZL, and LO performed the sample preparation. YLi and ML took charge of library construction and sequencing. LZho, LZen, and MW supported data mining and figure drawing. ZX and XY designed the database. All authors read and approved the final version of the manuscript.

## Funding

This work was supported by the Shenzhen Science and Technology Program: KCXFZ20201221173007020.

## Conflict of interest

LS, LZho, YLiu, XZhao, MW, XZhan, YLi, ML, LZen, and QY were employed by GeneMind Biosciences Company Limited. JF, JC, ZL, LO, ZX, XY, and LX were employed by the State Environmental Protection Key Laboratory of Drinking Water Source.

## Publisher’s note

All claims expressed in this article are solely those of the authors and do not necessarily represent those of their affiliated organizations, or those of the publisher, the editors and the reviewers. Any product that may be evaluated in this article, or claim that may be made by its manufacturer, is not guaranteed or endorsed by the publisher.

## Footnotes

^1^ http://www.fwalagedb.com/

^2^ https://gephi.org
