# Supplementary material for: Evaluation of environmental factors and microbial community structure in an important drinking-water reservoir across seasons
Source: Front Microbiol. 2023 Feb 14;14:1091818. doi: 10.3389/fmicb.2023.1091818 (PMC9971975; doi:10.3389/fmicb.2023.1091818)
Supplement: Supplementary file 1 [file Data_Sheet_1.docx]

**Supplementary Data**

**Evaluation of environmental factors and microbial community structure in an important drinking-water reservoir across seasons**

Jie Feng^1†^, Letian Zhou^2†^, Xiaochao Zhao^2†^, Jianyi Chen^1^, Zhi Li^1^, Yongfeng Liu^2^, Lei Ou^1^, Zixin Xie^1^, Miao Wang^2^, Xue Yin^1^, Xin Zhang^2^, Yan Li^2^, MingJie Luo^2^, Lidong Zeng^2^, Qin Yan^2^, Linshen Xie^1*^ and Lei Sun^2*^

^1^State Environmental Protection Key Laboratory of Drinking Water Source Management and Technology, Shenzhen Academy of Environmental Sciences, Shenzhen, 518001, China

^2^ GeneMind Biosciences Company Limited, Shenzhen, 518023, China

**Supplementary tables**

**Table S1** Sample information and data status.

**Table S2** Algal toxin gene information.

**Table S3** Relative abundance and diversity of algae community.

**Table S4** The relative abundance of algae in species level.

**Table S5** The BLAST result table.

**Figure Legends**

**Figure S1** Location of the investigated reservoirs in eastern China.

**Figure S2** Spearman correlations between sediment samples and water samples. S stands for sediment, W stands for water, QSK indicates the water intake, and KZ indicates the center of the reservoir.

**Figure S3** Histogram of the relative abundances of algae at the species level. The 20 most abundant species are shown, and ‘Other’ indicates species with relative abundances less than 0.006% across all samples.

**Figure S4** Histogram of the relative abundances of benthic cyanobacteria at the species level. Top five species in every sample are shown.

**Figure S5** Functional differences between sediment and water samples in the top 20 significant KEGG pathways. Green modules indicate sediment samples, and blue modules indicate water samples.

**Figure S6** Correlation heatmap for microbes and other environmental factors. The R-value is shown in the color gradient. * Indicates p ≤ 0.05, ** indicates p ≤ 0.01, and *** indicates p ≤ 0.001. PI: permanganate index, TN: total nitrogen, NH3.N: ammonia nitrogen, and TP: total phosphorus.

**Figure S7** Heatmap of the relative abundances of algal toxin gene clusters in all samples. The abscissa shows the sample information, and the ordinate shows the type of algal toxin; redder colors indicate higher abundances of algal toxin gene clusters.

**Figure S8** Abundances of ARGs according to metagenomic sequences. Source species are shown on the left, and types of ARGs are shown on the right. (a) Sediment samples; (b) water samples.

Table S1 Sample information and data status.

| Sample Name | Collection Date (D/M/Y) | Resource | Location | Raw Reads(M) |
| --- | --- | --- | --- | --- |
| S_QSK201020 | 20 October 2020 | sediment | Water-intake | 43.92 |
| S_KZ201103 | 3 November 2020 | sediment | internal | 37.62 |
| S_QSK201103 | 3 November 2020 | sediment | Water-intake | 94.52 |
| S_KZ201130 | 30 November 2020 | sediment | internal | 38.59 |
| S_QSK201130 | 30 November 2020 | sediment | Water-intake | 80.48 |
| S_KZ201214 | 14 December 2020 | sediment | internal | 87.92 |
| S_QSK201214 | 14 December 2020 | sediment | Water-intake | 39.80 |
| S_KZ201229 | 29 December 2020 | sediment | internal | 38.87 |
| S_QSK201229 | 29 December 2020 | sediment | Water-intake | 38.87 |
| W_QSK201020 | 20 October 2020 | Water | Water-intake | 30.19 |
| W_KZ201103 | 3 November 2020 | Water | internal | 44.06 |
| W_QSK201103 | 3 November 2020 | Water | Water-intake | 53.78 |
| W_KZ201116 | 16 November 2020 | Water | internal | 46.00 |
| W_QSK201116 | 16 November 2020 | Water | Water-intake | 52.58 |
| W_KZ201123 | 23 November 2020 | Water | internal | 56.71 |
| W_QSK201123 | 23 November 2020 | Water | Water-intake | 48.58 |
| W_KZ201130 | 30 November 2020 | Water | internal | 48.89 |
| W_QSK201130 | 30 November 2020 | Water | Water-intake | 43.44 |
| W_KZ201207 | 7 December 2020 | Water | internal | 46.52 |
| W_QSK201207 | 7 December 2020 | Water | Water-intake | 50.52 |
| W_KZ201214 | 14 December 2020 | Water | internal | 50.46 |
| W_QSK201214 | 14 December 2020 | Water | Water-intake | 91.64 |
| W_KZ201229 | 29 December 2020 | Water | internal | 64.24 |
| W_QSK201229 | 29 December 2020 | Water | Water-intake | 59.78 |
| W_KZ210104 | 4 January 2021 | Water | internal | 43.11 |
| W_QSK210104 | 4 January 2021 | Water | Water-intake | 50.27 |

Table S2 Algal toxin gene information.

| **Gene Cluster Accession Number** | **Species** | **Algal toxin** |
| --- | --- | --- |
| JF803645.1 | Anabaena sp. 37 | anatoxin-a |
| AJ536156.1 | Anabaena sp. 90 | microcystin |
| KM245023.1 | Cuspidothrix issatschenkoi LBRI48 | anatoxin-a |
| KM245024.1 | Cuspidothrix issatschenkoi RM-6 | anatoxin-a |
| EU140798.1 | Cylindrospermopsis raciborskii AWT205 | cylindrospermopsin |
| KJ139742.1 | Cylindrospermopsis raciborskii CHAB358 | cylindrospermopsin |
| KX891213.1 | Fischerella sp. CENA161 | microcystin cyclic heptapeptide |
| KC699835.1 | Nostoc sp. 152 | microcystin |
| MF668122.1 | Nostoc sp. CENA543 | nodularin |
| FJ477836.2 | Oscillatoria sp. PCC 6506 | anatoxin-a and homoanatoxin-a |
| FJ418586.4 | Oscillatoria sp. PCC 6506 | cylindrospermopsin |
| MK870090.1 | Phormidium sp. LP904c | microcystin |
| KX950803.1 | Phormidium sp. LP904e | microcystin |
| AJ441056.1 | *Planktothrix agardhii* | microcystin |
| JN873921.1 | Raphidiopsis curvata CHAB1150 | cylindrospermopsin |
| KJ139745.1 | Raphidiopsis curvata HB1 | cylindrospermopsin |

Table S3 Relative abundance and diversity of alage community.

| Samples name | observed_species | Chao1 | ACE | Shannon | Simpson | Coverage |
| --- | --- | --- | --- | --- | --- | --- |
| S_KZ201103 | 4,595.00 | 5,225.07 | 4,937.78 | 6.16 | 0.01 | 99.96% |
| S_KZ201130 | 4,597.00 | 5,240.55 | 4,962.24 | 6.26 | 0.01 | 99.96% |
| S_KZ201214 | 4,254.00 | 4,392.08 | 4,325.07 | 5.17 | 0.03 | 99.98% |
| S_KZ201229 | 4,238.00 | 4,343.06 | 4,300.40 | 6.06 | 0.01 | 99.99% |
| S_QSK201020 | 4,133.00 | 4,269.28 | 4,209.26 | 5.12 | 0.06 | 99.98% |
| S_QSK201103 | 4,191.00 | 4,252.65 | 4,235.76 | 5.15 | 0.04 | 99.99% |
| S_QSK201130 | 4,155.00 | 4,233.65 | 4,200.23 | 5.10 | 0.04 | 99.99% |
| S_QSK201214 | 4,240.00 | 4,346.74 | 4,290.42 | 6.05 | 0.01 | 99.99% |
| S_QSK201229 | 4,584.00 | 5,190.02 | 4,911.96 | 6.17 | 0.01 | 99.96% |
| W_KZ201103 | 4,243.00 | 4,375.27 | 4,327.48 | 4.59 | 0.05 | 99.98% |
| W_KZ201116 | 4,263.00 | 4,420.57 | 4,359.23 | 4.49 | 0.07 | 99.98% |
| W_KZ201123 | 4,236.00 | 4,330.44 | 4,298.40 | 4.65 | 0.05 | 99.98% |
| W_KZ201130 | 4,251.00 | 4,380.41 | 4,323.08 | 4.57 | 0.06 | 99.98% |
| W_KZ201207 | 4,262.00 | 4,511.38 | 4,356.67 | 4.58 | 0.06 | 99.98% |
| W_KZ201214 | 4,296.00 | 4,447.87 | 4,387.45 | 4.48 | 0.06 | 99.98% |
| W_KZ201229 | 4,203.00 | 4,280.00 | 4,263.93 | 4.51 | 0.06 | 99.98% |
| W_KZ210104 | 4,213.00 | 4,360.79 | 4,305.22 | 4.78 | 0.03 | 99.98% |
| W_QSK201020 | 4,140.00 | 4,245.01 | 4,194.03 | 4.38 | 0.07 | 99.99% |
| W_QSK201103 | 4,194.00 | 4,275.57 | 4,250.61 | 4.60 | 0.05 | 99.98% |
| W_QSK201116 | 4,186.00 | 4,284.45 | 4,243.83 | 4.49 | 0.07 | 99.99% |
| W_QSK201123 | 4,279.00 | 4,431.72 | 4,362.51 | 4.56 | 0.05 | 99.98% |
| W_QSK201130 | 4,224.00 | 4,388.07 | 4,304.94 | 4.53 | 0.06 | 99.98% |
| W_QSK201207 | 4,314.00 | 4,585.32 | 4,420.12 | 4.63 | 0.05 | 99.98% |
| W_QSK201214 | 4,222.00 | 4,370.61 | 4,295.64 | 4.75 | 0.04 | 99.98% |
| W_QSK201229 | 4,214.00 | 4,374.59 | 4,294.83 | 4.39 | 0.07 | 99.98% |
| W_QSK210104 | 4,215.00 | 4,335.74 | 4,284.56 | 4.58 | 0.04 | 99.98% |

Shannon, Simpson: diversity indice. ACE, Chao: community richness indice.

Coverage: sampling depth.

**Table S4** The relative abundance of algae in species level.

| species | S_KZ201103 | S_KZ201130 | S_KZ201214 | S_KZ201229 | S_QSK201020 | S_QSK201103 | S_QSK201130 | S_QSK201214 | S_QSK201229 | W_KZ201103 | W_KZ201116 | W_KZ201123 | W_KZ201130 | W_KZ201207 | W_KZ201214 | W_KZ201229 | W_KZ210104 | W_QSK201020 | W_QSK201103 | W_QSK201116 | W_QSK201123 | W_QSK201130 | W_QSK201207 | W_QSK201214 | W_QSK201229 | W_QSK210104 |
| --- | --- | --- | --- | --- | --- | --- | --- | --- | --- | --- | --- | --- | --- | --- | --- | --- | --- | --- | --- | --- | --- | --- | --- | --- | --- | --- |
| *Chara braunii* | 5.36479% | 5.54094% | 22.09600% | 7.39626% | 11.83403% | 26.96903% | 28.11941% | 7.34394% | 5.26226% | 23.40802% | 21.59389% | 34.96446% | 29.65287% | 31.85271% | 43.30815% | 26.84700% | 34.43804% | 9.37297% | 22.03559% | 20.82198% | 30.45626% | 25.84057% | 33.38260% | 19.32702% | 29.29119% | 34.21280% |
| *Euglena gracilis* | 4.48603% | 4.36172% | 4.19563% | 5.47814% | 7.07943% | 4.29446% | 4.46930% | 5.21650% | 4.26398% | 11.31727% | 10.19033% | 19.54164% | 15.28351% | 16.65903% | 21.63470% | 12.11834% | 15.99946% | 4.64019% | 10.74848% | 10.08066% | 15.69172% | 14.12903% | 17.59241% | 13.47464% | 14.66919% | 15.69896% |
| *Volvox africanus* | 0.61941% | 0.59065% | 0.57650% | 0.70290% | 1.04726% | 0.59866% | 0.62797% | 0.68553% | 0.59804% | 3.40001% | 2.56817% | 7.58038% | 5.57855% | 4.76304% | 6.67343% | 2.81062% | 6.30260% | 1.29124% | 3.71356% | 2.52353% | 5.57814% | 4.83386% | 4.82290% | 3.64677% | 2.71145% | 3.37605% |
| Chlamydomonas sp. UWO 241 | 2.07002% | 1.86805% | 1.67243% | 2.35946% | 3.41062% | 1.87318% | 1.99332% | 2.51681% | 1.99090% | 3.82295% | 2.45744% | 6.07275% | 4.13473% | 4.62828% | 6.86970% | 1.75202% | 5.65534% | 1.37324% | 3.33539% | 2.99289% | 5.42933% | 4.27059% | 5.40455% | 4.59853% | 4.26658% | 5.33588% |
| *Haematococcus lacustris* | 3.95749% | 3.47963% | 12.80235% | 4.76778% | 8.56644% | 14.40889% | 15.54442% | 4.83090% | 3.74439% | 3.80378% | 3.86005% | 4.57908% | 4.62135% | 4.65489% | 4.55196% | 3.79175% | 4.00632% | 2.55503% | 4.53434% | 4.03389% | 4.20433% | 4.78469% | 4.24821% | 3.64131% | 3.45655% | 3.76084% |
| *Botryococcus braunii* | 0.49053% | 0.49091% | 0.44904% | 0.49998% | 1.09736% | 0.45100% | 0.47594% | 0.48533% | 0.52036% | 1.99178% | 1.32959% | 3.23248% | 2.11112% | 2.37864% | 3.57856% | 1.06699% | 3.03908% | 0.58296% | 1.86204% | 1.55109% | 2.70252% | 1.96766% | 2.55595% | 1.88442% | 2.10596% | 3.19010% |
| Chlorella sp. KRBP | 3.64526% | 3.19908% | 2.96007% | 3.97204% | 8.38079% | 3.54032% | 3.68483% | 4.04714% | 3.46042% | 2.80969% | 2.80956% | 3.21085% | 3.02243% | 3.17039% | 2.76210% | 2.91946% | 2.72048% | 1.98497% | 3.07651% | 2.97527% | 2.95177% | 3.10746% | 2.81773% | 2.61207% | 2.40192% | 2.46696% |
| *Volvox reticuliferus* | 0.57211% | 0.52588% | 0.50839% | 0.63294% | 0.79020% | 0.54048% | 0.58077% | 0.62675% | 0.54145% | 1.69899% | 1.34822% | 3.18485% | 2.47085% | 2.33202% | 3.08708% | 1.48714% | 2.68825% | 0.73043% | 1.75808% | 1.36035% | 2.52349% | 2.22929% | 2.29937% | 1.73291% | 1.64688% | 1.79281% |
| *Cyclotella cryptica* | 0.41374% | 0.41268% | 0.38547% | 0.42693% | 2.60795% | 0.39685% | 0.61716% | 0.45694% | 0.37092% | 2.71157% | 6.66744% | 2.97130% | 3.31964% | 1.92093% | 2.97901% | 2.13599% | 2.53809% | 0.77607% | 2.75004% | 3.59522% | 3.00831% | 3.04617% | 2.10144% | 1.62855% | 2.82192% | 2.51867% |
| *Cyanobium gracile* | 0.05261% | 0.04611% | 0.04219% | 0.04424% | 0.78250% | 0.05119% | 0.08272% | 0.06556% | 0.03575% | 3.11534% | 2.24422% | 2.92506% | 2.71386% | 1.37997% | 1.33052% | 1.19337% | 1.20585% | 2.83543% | 3.31489% | 2.87129% | 3.51681% | 2.47124% | 1.29273% | 1.06844% | 1.06689% | 1.42285% |
| *Dunaliella salina* | 0.59497% | 0.59894% | 0.60971% | 0.65069% | 1.13136% | 0.62489% | 0.62312% | 0.63781% | 0.60730% | 1.49372% | 1.49319% | 2.23467% | 1.94668% | 1.93051% | 2.54849% | 1.31246% | 2.03527% | 0.81674% | 1.43771% | 1.55563% | 2.10012% | 1.90546% | 2.08620% | 1.46903% | 1.80487% | 2.03223% |
| Halamphora sp. AAB | 2.13300% | 1.78878% | 1.71279% | 1.94513% | 3.51852% | 2.00106% | 2.23664% | 2.11061% | 2.13932% | 2.22412% | 2.15148% | 1.94748% | 2.05123% | 1.97245% | 1.62085% | 2.75224% | 2.69122% | 1.39573% | 2.28434% | 2.03731% | 1.98336% | 2.03444% | 1.86589% | 1.27056% | 1.88752% | 2.01154% |
| Eudorina sp. 2006-703-Eu-15 | 1.90580% | 1.72116% | 1.53791% | 2.45513% | 2.19970% | 1.73029% | 1.80578% | 2.55977% | 1.86744% | 1.39650% | 1.29037% | 1.82169% | 1.53517% | 1.55092% | 1.90494% | 1.14788% | 1.65907% | 1.05151% | 1.41869% | 1.41793% | 1.76718% | 1.53978% | 1.55683% | 1.52741% | 1.63810% | 1.65407% |
| *Tribonema minus* | 1.85478% | 1.62739% | 1.49027% | 2.42864% | 2.09316% | 1.73251% | 1.82726% | 2.53691% | 1.84454% | 1.17274% | 1.34671% | 1.78714% | 1.94059% | 1.55113% | 1.73184% | 1.00038% | 1.33955% | 0.88089% | 1.17938% | 1.45146% | 1.63919% | 2.15976% | 1.41890% | 1.48019% | 1.41485% | 1.44022% |
| *Chlamydomonas incerta* | 2.85100% | 2.42994% | 2.17025% | 3.74134% | 3.27937% | 2.46464% | 2.66240% | 3.96299% | 2.69286% | 1.37349% | 1.35451% | 1.72119% | 1.48036% | 1.42063% | 1.60021% | 1.21014% | 1.55622% | 1.38118% | 1.41851% | 1.55279% | 1.76249% | 1.50479% | 1.35774% | 1.75665% | 1.86714% | 1.48295% |
| Pseudanabaena sp. ABRG5-3 | 0.05899% | 0.05181% | 0.05356% | 0.05092% | 0.14190% | 0.06420% | 0.06036% | 0.04522% | 0.05273% | 7.52071% | 2.25917% | 1.60324% | 2.86023% | 0.70659% | 0.62641% | 2.39435% | 2.39456% | 3.95473% | 6.29815% | 1.77933% | 2.76233% | 1.70215% | 0.72787% | 0.40240% | 3.01684% | 5.86857% |
| *Skeletonema costatum* | 0.23490% | 0.17175% | 0.18398% | 0.15894% | 1.70010% | 0.18129% | 0.47719% | 0.22282% | 0.16977% | 3.59625% | 9.41503% | 1.50397% | 4.87491% | 1.72464% | 2.43107% | 3.69796% | 5.33056% | 0.47186% | 3.53445% | 4.25209% | 1.85169% | 3.90788% | 2.76526% | 2.13705% | 7.88063% | 4.89724% |
| Chlamydomonas sp. 3222 | 1.95895% | 1.59475% | 1.47708% | 2.15988% | 2.98514% | 1.72658% | 1.87595% | 2.25104% | 1.83837% | 1.34800% | 1.18506% | 1.47029% | 1.26943% | 1.24883% | 1.26334% | 1.24477% | 1.51417% | 1.07895% | 1.41740% | 1.27417% | 1.46384% | 1.33533% | 1.11621% | 1.09970% | 1.14175% | 1.26012% |
| *Gloeobacter violaceus* | 0.00106% | 0.89530% | 0.83258% | 0.95726% | 1.09736% | 0.91596% | 0.94965% | 0.00201% | 0.00180% | 1.21063% | 0.94822% | 1.07947% | 1.07901% | 0.71234% | 0.61609% | 0.65615% | 0.58350% | 0.00198% | 1.24179% | 1.09006% | 1.20663% | 0.98680% | 0.66479% | 0.54787% | 0.49718% | 0.00624% |
| Chlorella sp. ArM0029B | 0.83944% | 0.79375% | 0.70010% | 0.98066% | 1.15176% | 0.76990% | 0.79986% | 1.02190% | 0.82337% | 0.87228% | 0.79979% | 1.06255% | 0.89548% | 0.81410% | 0.98119% | 0.71546% | 0.86988% | 0.56047% | 0.88837% | 0.90103% | 1.01352% | 0.89167% | 0.87545% | 0.66273% | 0.68714% | 0.80879% |
| *Synechococcus elongatus* | 0.28114% | 0.23885% | 0.22162% | 0.24433% | 0.51638% | 0.25353% | 0.27436% | 0.24417% | 0.26931% | 1.16845% | 0.86350% | 1.05522% | 0.98095% | 0.57311% | 0.54658% | 0.49111% | 0.50328% | 1.02340% | 1.22388% | 1.01979% | 1.21255% | 0.89327% | 0.54132% | 0.43126% | 0.42331% | 0.54928% |
| *Asterionellopsis glacialis* | 0.22454% | 0.21165% | 0.21423% | 0.22427% | 0.68548% | 0.23439% | 0.37621% | 0.25824% | 0.19755% | 0.94581% | 1.28863% | 1.04388% | 1.23086% | 1.08639% | 1.31884% | 1.07956% | 1.13157% | 0.42656% | 0.93601% | 0.98930% | 1.07680% | 1.11007% | 1.12991% | 0.67216% | 1.25205% | 1.22090% |
| *Micractinium conductrix* | 1.28081% | 1.13130% | 0.99188% | 1.59766% | 1.75631% | 1.13417% | 1.20737% | 1.65694% | 1.22309% | 0.86596% | 0.86675% | 0.98421% | 0.87701% | 0.81751% | 0.89785% | 0.71640% | 0.85776% | 0.76019% | 0.92271% | 0.95084% | 1.02087% | 0.86606% | 0.77147% | 0.83722% | 0.90128% | 0.82694% |
| *Nannochloropsis limnetica* | 0.49266% | 0.43729% | 8.75785% | 0.91277% | 0.98016% | 10.32005% | 10.49112% | 0.78350% | 0.36140% | 0.69521% | 0.66176% | 0.97461% | 0.79012% | 0.81559% | 1.00495% | 0.72913% | 0.86919% | 0.87560% | 0.66014% | 0.65593% | 0.86716% | 0.71695% | 0.88761% | 0.92232% | 0.85772% | 0.92079% |
| Snowella sp. | 0.96672% | 0.88701% | 28.60355% | 1.97830% | 1.04590% | 33.44255% | 37.50339% | 1.84785% | 0.22456% | 0.94288% | 0.87195% | 0.95961% | 0.90665% | 0.66252% | 0.87410% | 1.00705% | 0.80748% | 1.73896% | 0.89095% | 0.89175% | 0.85716% | 0.77984% | 0.88221% | 0.93820% | 1.38935% | 0.76235% |
| *Tetradesmus acuminatus* | 0.49053% | 0.45905% | 0.45188% | 0.49098% | 1.51581% | 0.49151% | 0.53295% | 0.52979% | 0.48461% | 0.81521% | 0.76165% | 0.94216% | 0.81570% | 0.93225% | 0.98587% | 0.69326% | 0.80154% | 0.56411% | 0.85051% | 0.81239% | 0.92411% | 0.84410% | 0.85713% | 0.74560% | 0.74047% | 0.80118% |
| *Chlamydomonas applanata* | 0.61543% | 0.56163% | 2.21493% | 0.69596% | 0.89720% | 0.59146% | 0.65653% | 0.73552% | 0.60653% | 0.72272% | 0.61236% | 0.93274% | 0.80271% | 0.83262% | 1.02267% | 0.54576% | 0.79125% | 0.45433% | 0.71387% | 0.71938% | 0.93411% | 0.74645% | 0.88568% | 0.70450% | 0.70850% | 0.81387% |
| *Ochromonas danica* | 0.24394% | 0.22123% | 0.24015% | 0.24870% | 0.37855% | 0.26538% | 0.27685% | 0.24467% | 0.21993% | 0.67017% | 0.61344% | 0.83434% | 0.78626% | 0.90926% | 1.20337% | 0.65212% | 0.99809% | 0.35877% | 0.65146% | 0.59873% | 0.81592% | 0.77458% | 0.82530% | 0.54117% | 0.83453% | 1.14636% |
| Pseudanabaena sp. PCC 7367 | 0.00186% | 0.10310% | 0.09347% | 0.00154% | 0.15324% | 0.09667% | 0.10259% | 0.10701% | 0.00206% | 0.00451% | 0.00368% | 0.83224% | 0.00589% | 0.59205% | 0.99210% | 0.00373% | 0.00274% | 0.25494% | 0.00480% | 0.00625% | 1.17622% | 0.00572% | 1.16830% | 0.00050% | 0.00149% | 0.00449% |
| *Auxenochlorella pyrenoidosa* | 1.01615% | 0.86343% | 0.76424% | 1.20879% | 1.29910% | 0.90179% | 0.94468% | 1.26054% | 0.94915% | 0.63814% | 0.63749% | 0.71081% | - | - | - | - | - | - | - | - | - | - | - | - | - | - |
| Pseudanabaena sp. FACHB-2040 | 0.29735% | 0.25387% | 0.23845% | 0.24330% | 0.54970% | 0.25691% | 0.27238% | 0.24040% | 0.26700% | 0.75656% | 0.60781% | 0.68324% | 0.66425% | 0.39385% | 0.38808% | 0.37279% | 0.38488% | 0.61702% | 0.80416% | 0.64722% | 0.76652% | 0.58248% | 0.39683% | 0.30193% | 0.34166% | 0.40566% |
| *Chlorella variabilis* | 1.02518% | 0.90669% | 0.82291% | 1.31244% | 1.27734% | 0.93712% | 1.00219% | 1.37961% | 1.00497% | 0.55422% | 0.52135% | 0.62462% | 0.55727% | 0.50221% | 0.55164% | 0.48427% | 0.53779% | 0.54063% | 0.57833% | 0.59627% | 0.67201% | 0.58888% | 0.48596% | 0.59170% | 0.62205% | 0.50850% |
| Monoraphidium sp. 549 | 0.26839% | 0.24222% | 0.25016% | 0.23661% | 1.07763% | 0.26178% | 0.28579% | 0.24744% | 0.28809% | 0.56505% | 0.49340% | 0.62113% | 0.57879% | 0.67551% | 0.80244% | 0.50694% | 0.67469% | 0.31182% | 0.58775% | 0.51103% | 0.62914% | 0.52896% | 0.59264% | 0.43242% | 0.58661% | 0.79143% |
| *Raphidiopsis curvata* | 0.00159% | 0.00104% | 0.00887% | 0.00231% | 0.00159% | 0.00825% | 0.00795% | 0.00176% | 0.00772% | 0.21294% | 0.92222% | 0.61973% | 1.52015% | 0.07494% | 0.09210% | 0.08167% | 0.12639% | 0.40903% | 0.56652% | 0.60782% | 1.03189% | 0.80179% | 0.10128% | 0.00058% | 0.18897% | 0.16644% |
| *Klebsormidium nitens* | 0.76450% | 0.69608% | 0.64518% | 0.78443% | 0.76436% | 0.70083% | 0.73341% | 0.81365% | 0.74826% | 0.53528% | 0.49621% | 0.61083% | 0.57473% | 0.54288% | 0.56332% | 0.46782% | 0.54213% | 0.35216% | 0.54897% | 0.56501% | 0.57443% | 0.55961% | 0.53997% | 0.44210% | 0.47830% | 0.55045% |
| Pseudanabaena sp. SR411 | 0.04252% | 0.03445% | 0.04207% | 0.03755% | 0.08092% | 0.04717% | 0.04931% | 0.04296% | 0.04501% | 2.38472% | 0.81387% | 0.61066% | 1.01140% | 0.31380% | 0.31233% | 0.87570% | 0.80885% | 1.19468% | 2.04116% | 0.66048% | 1.00842% | 0.64377% | 0.34879% | 0.18921% | 1.05166% | 1.89817% |
| *Nannochloropsis gaditana* | 0.22534% | 0.20932% | 0.20308% | 0.22504% | 0.41732% | 0.21767% | 0.21822% | 0.22709% | 0.20912% | 0.43061% | 0.35428% | 0.60316% | 0.45637% | 0.50839% | 0.66068% | 0.31985% | 0.62715% | 0.19873% | 0.41916% | 0.36708% | 0.51360% | 0.43017% | 0.51682% | 0.36668% | 0.44700% | 0.68684% |
| Synechococcus sp. WH 7803 | 0.01780% | 0.01140% | 0.01217% | 0.01260% | 0.14893% | 0.01523% | 0.01677% | 0.01256% | 0.01312% | 0.66723% | 0.46393% | 0.59862% | 0.53230% | 0.34425% | 0.30551% | 0.25510% | 0.25552% | 0.64843% | 0.69633% | 0.59721% | 0.72079% | 0.49009% | 0.32757% | 0.26868% | 0.24263% | 0.30752% |
| *Asterionella formosa* | 0.19398% | 0.18885% | 0.18466% | 0.18801% | 0.67188% | 0.19557% | 0.29523% | 0.21227% | 0.17440% | 0.59866% | 0.78419% | 0.56896% | 0.73774% | 0.60845% | 0.74305% | 0.60352% | 0.64178% | 0.30553% | 0.59828% | 0.59892% | 0.65159% | 0.64948% | 0.69739% | 0.43523% | 0.71629% | 0.74889% |
| *Marinobacter algicola* | 0.76025% | 0.60541% | 0.58333% | 0.66895% | 0.86070% | 0.70644% | 0.73950% | 0.70965% | 0.78453% | 0.49964% | 0.43879% | 0.48347% | 0.44277% | 0.41003% | 0.35751% | 0.46828% | 0.49939% | 0.35050% | 0.52515% | 0.45762% | 0.46318% | 0.49054% | 0.36808% | 0.29738% | 0.28155% | 0.37425% |
| Synechococcus sp. KORDI-100 | 0.01541% | 0.01995% | 0.01433% | 0.01492% | 0.11311% | 0.01597% | 0.01801% | 0.01583% | 0.01286% | 0.55422% | 0.38028% | 0.47021% | 0.40968% | 0.24483% | 0.23600% | 0.17421% | 0.17302% | 0.51054% | 0.55913% | 0.49171% | 0.56218% | 0.36293% | 0.22571% | 0.18863% | 0.17158% | 0.22381% |
| *Spirulina major* | 0.23650% | 0.21035% | 0.19797% | 0.20292% | 0.36223% | 0.21196% | 0.22890% | 0.22307% | 0.21349% | 0.53483% | 0.44832% | 0.46445% | 0.46957% | 0.28442% | 0.28059% | 0.27963% | 0.28135% | 0.39415% | 0.54934% | 0.43754% | 0.54463% | 0.40067% | 0.29208% | 0.20525% | 0.24859% | 0.29249% |
| Pseudanabaena sp. FACHB-1998 | 0.05979% | 0.04637% | 0.04105% | 0.04218% | 0.11583% | 0.04908% | 0.05328% | 0.04848% | 0.04141% | 1.65275% | 0.60889% | 0.46410% | 0.72901% | 0.24866% | 0.24320% | 0.64296% | 0.59653% | 0.87593% | 1.48073% | 0.50952% | 0.72039% | 0.49306% | 0.25214% | 0.14083% | 0.74676% | 1.36685% |
| Pseudanabaena sp. FACHB-1050 | 0.04358% | 0.04559% | 0.04048% | 0.03961% | 0.07594% | 0.04284% | 0.04757% | 0.04019% | 0.04347% | 1.70643% | 0.66154% | 0.44770% | 0.78647% | 0.24674% | 0.24515% | 0.63053% | 0.55493% | 0.87460% | 1.46319% | 0.53641% | 0.76285% | 0.49580% | 0.24944% | 0.14422% | 0.77392% | 1.29309% |
| Synechococcus sp. KORDI-52 | 0.01408% | 0.00596% | 0.00796% | 0.00849% | 0.12535% | 0.00888% | 0.01217% | 0.01331% | 0.00926% | 0.53325% | 0.37118% | 0.43968% | 0.40866% | 0.21034% | 0.20368% | 0.16225% | 0.17164% | 0.52774% | 0.58240% | 0.48470% | 0.53605% | 0.35584% | 0.20160% | 0.18938% | 0.15468% | 0.19942% |
| *Auxenochlorella protothecoides* | 0.71268% | 0.63831% | 0.57878% | 0.86802% | 0.74306% | 0.64752% | 0.67578% | 0.93448% | 0.70993% | 0.38008% | 0.35775% | 0.42833% | 0.38369% | 0.34999% | 0.36880% | 0.34531% | 0.34009% | 0.28602% | 0.41750% | 0.39000% | 0.43480% | 0.39335% | 0.34378% | 0.35361% | 0.35856% | 0.32918% |
| Synechococcus sp. WH 8109 | 0.01302% | 0.00803% | 0.00864% | 0.00592% | 0.08636% | 0.00941% | 0.01192% | 0.00879% | 0.00797% | 0.38166% | 0.27953% | 0.33953% | 0.29295% | 0.18160% | 0.16707% | 0.13648% | 0.12730% | 0.33926% | 0.41972% | 0.34018% | 0.41317% | 0.26162% | 0.14488% | 0.13860% | 0.12090% | 0.16293% |
| Chloroidium sp. CF | 0.30293% | 0.30128% | 0.26631% | 0.27982% | 0.32098% | 0.27902% | 0.28355% | 0.30697% | 0.29169% | 0.29347% | 0.26804% | 0.33325% | 0.31284% | 0.29145% | 0.32849% | 0.25355% | 0.26832% | 0.19641% | 0.28437% | 0.28128% | 0.34396% | 0.30622% | 0.30307% | 0.21683% | 0.26118% | 0.29269% |
| *Coccomyxa subellipsoidea* | 0.36299% | 0.35102% | 0.33703% | 0.42642% | 0.40236% | 0.37337% | 0.36304% | 0.41800% | 0.38841% | 0.27745% | 0.25374% | 0.33202% | 0.30655% | 0.29805% | 0.30026% | 0.25650% | 0.30261% | 0.20766% | 0.28270% | 0.27806% | 0.30845% | 0.29250% | 0.28494% | 0.24784% | 0.25538% | 0.28078% |
| *Leptolyngbya foveolarum* | 0.16342% | 0.14663% | 0.13509% | 0.13528% | 0.26635% | 0.14639% | 0.15749% | 0.15072% | 0.15150% | 0.32865% | 0.26414% | 0.31545% | 0.30310% | 0.21885% | 0.19647% | 0.23709% | 0.23770% | 0.26321% | 0.33016% | 0.29207% | 0.34662% | 0.30759% | 0.20160% | 0.15233% | 0.18979% | 0.22361% |
| Pseudanabaena sp. BC1403 | 0.03906% | 0.03005% | 0.03445% | 0.03318% | 0.07163% | 0.03861% | 0.03391% | 0.03617% | 0.03627% | 0.95641% | 0.42384% | 0.30341% | 0.48276% | 0.20480% | 0.19044% | 0.41068% | 0.35837% | 0.51947% | 0.82854% | 0.36462% | 0.47583% | 0.32817% | 0.19562% | 0.11305% | 0.45577% | 0.76333% |
| *Microcoleus vaginatus* | 0.16874% | 0.16502% | 0.14464% | 0.14583% | 0.21172% | 0.14607% | 0.17165% | 0.15072% | 0.15871% | 0.35121% | 0.31940% | 0.30201% | 0.38674% | 0.24057% | 0.24223% | 0.25339% | 0.23518% | 0.23179% | 0.33773% | 0.32029% | 0.34458% | 0.29135% | 0.24211% | 0.15299% | 0.25008% | 0.25074% |
| Pseudanabaena sp. PCC 6802 | 0.19876% | 0.17668% | 0.17318% | 0.15817% | 0.22305% | 0.18404% | 0.24257% | 0.17886% | 0.18803% | 0.35211% | 0.30141% | 0.29033% | 0.37456% | 0.22396% | 0.20777% | 0.22886% | 0.22238% | 0.24998% | 0.36303% | 0.29700% | 0.33907% | 0.28701% | 0.22340% | 0.14447% | 0.24362% | 0.26517% |
| *Aphanizomenon flos-aquae* | 0.05474% | 0.06114% | 0.06527% | 0.06507% | 0.13646% | 0.05616% | 0.06881% | 0.06431% | 0.05402% | 0.31106% | 0.38245% | 0.28858% | 0.46002% | 0.20629% | 0.20757% | 0.23647% | 0.20707% | 0.21195% | 0.28861% | 0.35609% | 0.43787% | 0.39998% | 0.25793% | 0.12967% | 0.26498% | 0.26888% |
| Pseudanabaena sp. lw0831 | 0.04039% | 0.03964% | 0.03559% | 0.04141% | 0.07004% | 0.04294% | 0.04819% | 0.03642% | 0.03524% | 0.85829% | 0.37097% | 0.26171% | 0.42714% | 0.17798% | 0.14721% | 0.35587% | 0.32226% | 0.43548% | 0.72956% | 0.30647% | 0.41847% | 0.31491% | 0.17517% | 0.10296% | 0.42878% | 0.68782% |
| Nitzschia sp. Nitz4 | 0.09247% | 0.07746% | 0.08858% | 0.07484% | 0.15346% | 0.08620% | 0.09390% | 0.08164% | 0.07614% | 0.23910% | 0.23077% | 0.25369% | 0.26777% | 0.24440% | 0.31291% | 0.17918% | 0.33917% | 0.09887% | 0.23377% | 0.20949% | 0.24659% | 0.24607% | 0.23709% | 0.17036% | 0.27724% | 0.37796% |
| *Prochlorococcus marinus* | 0.03694% | 0.03160% | 0.03013% | 0.02906% | 0.07888% | 0.02813% | 0.03006% | 0.03040% | 0.02984% | 0.24407% | 0.22297% | 0.24426% | 0.24910% | 0.19203% | 0.20076% | 0.18477% | 0.18170% | 0.18649% | 0.25205% | 0.24586% | 0.27742% | 0.22366% | 0.20565% | 0.14232% | 0.18797% | 0.20430% |
| Pseudanabaena sp. UWO311 | 0.04013% | 0.04171% | 0.03298% | 0.03446% | 0.06098% | 0.03765% | 0.04024% | 0.03869% | 0.04064% | 0.75543% | 0.33565% | 0.24147% | 0.36765% | 0.14477% | 0.14059% | 0.32978% | 0.30512% | 0.40109% | 0.66641% | 0.28147% | 0.36744% | 0.25568% | 0.16436% | 0.08650% | 0.36866% | 0.62420% |
| *Spirulina subsalsa* | 0.11347% | 0.11217% | 0.09392% | 0.09259% | 0.21648% | 0.09604% | 0.11774% | 0.10199% | 0.09877% | 0.29504% | 0.28386% | 0.23990% | 0.32421% | 0.18649% | 0.18167% | 0.19812% | 0.16524% | 0.21394% | 0.29895% | 0.26612% | 0.31049% | 0.25522% | 0.20314% | 0.12049% | 0.20768% | 0.22595% |
| *Microcystis wesenbergii* | 0.02232% | 0.01865% | 0.01933% | 0.01877% | 0.08070% | 0.01936% | 0.06049% | 0.02462% | 0.02186% | 0.16489% | 0.31441% | 0.20448% | 0.25214% | 0.10794% | 0.07516% | 0.08633% | 0.05188% | 0.08002% | 0.16434% | 0.29397% | 0.29354% | 0.21063% | 0.10880% | 0.11321% | 0.10632% | 0.07220% |
| *Chroomonas mesostigmatica* | 0.01674% | 0.02254% | 0.02183% | 0.02829% | 0.04987% | 0.01957% | 0.02000% | 0.02512% | 0.01775% | 0.13918% | 0.11419% | 0.19035% | 0.18230% | 0.22801% | 0.26638% | 0.16831% | 0.18467% | 0.05489% | 0.12907% | 0.10077% | 0.20291% | 0.16420% | 0.23324% | 0.13430% | 0.20652% | 0.17151% |
| Pseudanabaena sp. Roaring Creek | 0.03986% | 0.03497% | 0.03479% | 0.03241% | 0.07503% | 0.03205% | 0.03565% | 0.03492% | 0.03550% | 0.60949% | 0.26111% | 0.18355% | 0.28381% | 0.14498% | 0.15247% | 0.25386% | 0.22261% | 0.32041% | 0.53309% | 0.20589% | 0.28517% | 0.18364% | 0.10996% | 0.06847% | 0.28966% | 0.49484% |
| *Anabaena azotica* | 0.08796% | 0.09300% | 0.09279% | 0.08770% | 0.16865% | 0.09160% | 0.10060% | 0.09998% | 0.08566% | 0.21046% | 0.21755% | 0.17971% | 0.25600% | 0.17074% | 0.19550% | 0.21271% | 0.17393% | 0.11606% | 0.20533% | 0.19983% | 0.21414% | 0.19553% | 0.17999% | 0.09783% | 0.21116% | 0.19688% |
| Dolichospermum sp. DET69 | 0.03826% | 0.04093% | 0.04469% | 0.03755% | 0.11810% | 0.04083% | 0.05763% | 0.04622% | 0.03755% | 0.17685% | 0.24204% | 0.17116% | 0.28340% | 0.14200% | 0.13903% | 0.16163% | 0.15999% | 0.13392% | 0.16730% | 0.20835% | 0.25272% | 0.22869% | 0.15723% | 0.07724% | 0.16843% | 0.17015% |
| *Scenedesmus vacuolatus* | 0.09194% | 0.09689% | 0.08323% | 0.09182% | 0.21852% | 0.09043% | 0.09539% | 0.08993% | 0.07974% | 0.15158% | 0.15926% | 0.16610% | 0.15530% | 0.18181% | 0.17856% | 0.15402% | 0.18010% | 0.11143% | 0.16600% | 0.14149% | 0.17188% | 0.16557% | 0.15395% | 0.12917% | 0.15684% | 0.18088% |
| *Cylindrospermum stagnale* | 0.06218% | 0.00233% | 0.06925% | 0.06430% | 0.00204% | 0.06843% | 0.07874% | 0.06808% | 0.06276% | 0.19399% | 0.19567% | 0.16488% | 0.00447% | 0.16669% | 0.16025% | 0.00295% | 0.14902% | 0.12102% | 0.00554% | 0.18240% | 0.20087% | 0.17838% | 0.17343% | 0.00099% | 0.00132% | 0.17230% |
| *Chlorogloea purpurea* | 0.07626% | 0.07305% | 0.06720% | 0.07381% | 0.14145% | 0.07256% | 0.07924% | 0.06556% | 0.07485% | 0.17730% | 0.13998% | 0.15109% | 0.15145% | 0.11177% | 0.10009% | 0.10154% | 0.10445% | 0.11176% | 0.17671% | 0.14319% | 0.16514% | 0.12967% | 0.11652% | 0.06798% | 0.09424% | 0.10068% |
| *Nostoc linckia* | 0.09301% | 0.00855% | 0.08471% | 0.08436% | 0.13170% | 0.00984% | 0.01056% | 0.08466% | 0.01003% | 0.18767% | 0.16273% | 0.14603% | 0.18352% | 0.12625% | 0.13961% | 0.00932% | 0.13279% | 0.10151% | 0.17154% | 0.16536% | 0.17392% | 0.16695% | 0.15703% | 0.00604% | 0.15932% | 0.15005% |
| *Microcystis novacekii* | 0.02232% | 0.02280% | 0.02126% | 0.01877% | 0.09974% | 0.02242% | 0.07713% | 0.01557% | 0.01826% | 0.12045% | 0.16685% | 0.13400% | 0.13703% | 0.06280% | 0.04751% | 0.04487% | 0.03017% | 0.03935% | 0.12261% | 0.16403% | 0.16290% | 0.11755% | 0.06559% | 0.06111% | 0.04107% | 0.04312% |
| *Nostoc commune* | 0.04385% | 0.04197% | 0.04389% | 0.04321% | 0.06528% | 0.04474% | 0.05080% | 0.04798% | 0.03395% | 0.13624% | 0.12048% | 0.12457% | 0.13480% | 0.11730% | 0.13650% | 0.11583% | 0.14947% | 0.08763% | 0.14071% | 0.11762% | 0.13861% | 0.11206% | 0.15433% | 0.06980% | 0.12670% | 0.13366% |
| Microcystis sp. LSC13-02 | 0.01966% | 0.01451% | 0.01876% | 0.01903% | 0.08546% | 0.02052% | 0.05167% | 0.01432% | 0.02006% | 0.11346% | 0.16750% | 0.12161% | 0.11612% | 0.06557% | 0.05160% | 0.04751% | 0.03245% | 0.04596% | 0.11319% | 0.15115% | 0.13963% | 0.11275% | 0.06984% | 0.05524% | 0.04869% | 0.04371% |
| *Nostoc piscinale* | 0.03906% | 0.04793% | 0.04719% | 0.04475% | 0.10246% | 0.04844% | 0.05241% | 0.04924% | 0.04579% | 0.11707% | 0.00195% | 0.10992% | 0.13947% | 0.00128% | 0.09327% | 0.11397% | 0.10056% | 0.00132% | 0.11559% | 0.11100% | 0.00184% | 0.00183% | 0.09762% | 0.06037% | 0.12123% | 0.10966% |
| *Anabaena catenula* | 0.04305% | 0.04767% | 0.04014% | 0.04218% | 0.08251% | 0.03956% | 0.04633% | 0.03567% | 0.04038% | 0.12564% | 0.13110% | 0.10957% | 0.13439% | 0.09708% | 0.09794% | 0.11319% | 0.10353% | 0.07605% | 0.11744% | 0.11687% | 0.13514% | 0.13058% | 0.10244% | 0.05260% | 0.10268% | 0.10127% |
| *Nostoc flagelliforme* | 0.04677% | 0.05207% | 0.05083% | 0.04321% | 0.07299% | 0.04717% | 0.05204% | 0.05074% | 0.05427% | 0.10444% | 0.09794% | 0.10503% | 0.11186% | 0.00170% | 0.08217% | 0.08462% | 0.08434% | 0.00132% | 0.09953% | 0.09508% | 0.10268% | 0.09399% | 0.09318% | 0.04871% | 0.08827% | 0.08839% |
| *Microcystis flos-aquae* | 0.01249% | 0.01399% | 0.01274% | 0.01157% | 0.03491% | 0.01597% | 0.04210% | 0.01130% | 0.01389% | 0.11098% | 0.14995% | 0.08811% | 0.11227% | 0.05833% | 0.04771% | 0.05450% | 0.03245% | 0.04199% | 0.09824% | 0.13107% | 0.12758% | 0.09879% | 0.06540% | 0.04623% | 0.05830% | 0.05542% |
| *Nostoc sphaeroides* | 0.03747% | 0.04352% | 0.00375% | 0.03884% | 0.07390% | 0.04569% | 0.04670% | 0.03944% | 0.00206% | 0.09068% | 0.08602% | 0.08392% | 0.10151% | 0.00809% | 0.08568% | 0.07732% | 0.10056% | 0.00628% | 0.09325% | 0.08334% | 0.09533% | 0.07913% | 0.09221% | 0.05119% | 0.08761% | 0.09737% |
| Geitlerinema sp. PCC 7407 | 0.03401% | 0.02539% | 0.02604% | 0.03343% | 0.04851% | 0.02708% | 0.02670% | 0.03291% | 0.02727% | 0.08166% | 0.05265% | 0.08165% | 0.08161% | 0.04237% | 0.04654% | 0.03649% | 0.03405% | 0.07242% | 0.08716% | 0.07463% | 0.10309% | 0.06724% | 0.04263% | 0.03448% | 0.03180% | 0.04273% |
| Synechococcus sp. CC9311 | 0.00266% | 0.00311% | 0.00398% | 0.00412% | 0.01609% | 0.00370% | 0.00460% | 0.00301% | 0.00386% | 0.08955% | 0.06392% | 0.08165% | 0.06598% | 0.04577% | 0.04342% | 0.03276% | 0.03268% | 0.07407% | 0.09103% | 0.07160% | 0.08880% | 0.05832% | 0.03337% | 0.03415% | 0.02633% | 0.03805% |
| Leptolyngbya sp. O-77 | 0.02764% | 0.03005% | 0.02763% | 0.03086% | 0.08750% | 0.02443% | 0.02732% | 0.02336% | 0.02598% | 0.06158% | 0.06457% | 0.05897% | 0.07146% | 0.03726% | 0.03524% | 0.04270% | 0.04045% | 0.05522% | 0.07183% | 0.05796% | 0.06696% | 0.05214% | 0.03530% | 0.03043% | 0.03097% | 0.04312% |
| *Halomicronema hongdechloris* | 0.02259% | 0.02306% | 0.01808% | 0.02238% | 0.02516% | 0.02306% | 0.02484% | 0.02261% | 0.02804% | 0.05233% | 0.03705% | 0.04414% | 0.04730% | 0.02555% | 0.02376% | 0.02438% | 0.03337% | 0.04431% | 0.05023% | 0.04622% | 0.05165% | 0.04231% | 0.02720% | 0.02158% | 0.02186% | 0.02712% |
| Synechococcus sp. PCC 7336 | 0.01754% | 0.01528% | 0.01387% | 0.01723% | 0.01745% | 0.01639% | 0.01962% | 0.01482% | 0.01415% | 0.03677% | 0.02817% | 0.03908% | 0.02639% | 0.01873% | 0.01675% | 0.01817% | 0.01988% | 0.02513% | 0.03915% | 0.02898% | 0.03817% | 0.02264% | 0.01852% | 0.01373% | 0.01391% | 0.01795% |
| *Planktothrix prolifica* | 0.01063% | 0.00984% | 0.01103% | 0.01337% | 0.01881% | 0.00994% | 0.01391% | 0.01156% | 0.00875% | 0.05211% | 0.03814% | 0.03629% | 0.04426% | 0.03683% | 0.03583% | 0.03602% | 0.03474% | 0.02182% | 0.04358% | 0.04281% | 0.04042% | 0.04414% | 0.02971% | 0.01580% | 0.03296% | 0.04039% |
| Cyanothece sp. PCC 7425 | 0.01408% | 0.01036% | 0.01444% | 0.01132% | 0.02924% | 0.01333% | 0.01441% | 0.01357% | 0.01080% | 0.04421% | 0.03532% | 0.03472% | 0.04020% | 0.02214% | 0.02376% | 0.01894% | 0.01691% | 0.03538% | 0.04136% | 0.02936% | 0.03817% | 0.03202% | 0.02373% | 0.01513% | 0.02054% | 0.02478% |
| Geminocystis sp. NIES-3709 | 0.00771% | 0.01062% | 0.00660% | 0.00849% | 0.02312% | 0.00698% | 0.01155% | 0.00779% | 0.00669% | 0.04466% | 0.03857% | 0.03455% | 0.04162% | 0.02682% | 0.02415% | 0.02438% | 0.01943% | 0.01984% | 0.04930% | 0.03409% | 0.04001% | 0.03499% | 0.02836% | 0.01745% | 0.03014% | 0.02029% |
| *Sphaerospermopsis kisseleviana* | 0.00797% | 0.01088% | 0.00762% | 0.00643% | 0.01655% | 0.00762% | 0.00907% | 0.01055% | 0.00746% | 0.04286% | 0.04312% | 0.03402% | 0.08831% | 0.03470% | 0.03544% | 0.02282% | 0.01554% | 0.02745% | 0.03324% | 0.04016% | 0.05757% | 0.05260% | 0.04534% | 0.01811% | 0.02633% | 0.03161% |
| Synechococcus sp. NIES-970 | 0.00691% | 0.00959% | 0.00534% | 0.00772% | 0.01111% | 0.00772% | 0.00633% | 0.00578% | 0.00669% | 0.04557% | 0.04399% | 0.03298% | 0.02761% | 0.02342% | 0.01967% | 0.03028% | 0.02651% | 0.02579% | 0.03970% | 0.03504% | 0.03307% | 0.03362% | 0.01948% | 0.01191% | 0.01805% | 0.02576% |
| Synechocystis sp. PCC 6714 | 0.00850% | 0.00803% | 0.00728% | 0.00823% | 0.03740% | 0.00571% | 0.01081% | 0.00653% | 0.00643% | 0.02887% | 0.02752% | 0.02373% | 0.02456% | 0.00915% | 0.00915% | 0.00994% | 0.00960% | 0.01257% | 0.03712% | 0.02595% | 0.02654% | 0.01669% | 0.01003% | 0.00595% | 0.00977% | 0.01151% |
| Synechococcus sp. JA-2-3Ba(2-13) | 0.01727% | 0.01684% | 0.01615% | 0.01415% | 0.02289% | 0.01396% | 0.01515% | 0.01733% | 0.01466% | 0.03045% | 0.03077% | 0.02338% | 0.03167% | 0.01554% | 0.01694% | 0.01428% | 0.01714% | 0.03009% | 0.03638% | 0.03106% | 0.03144% | 0.02561% | 0.01717% | 0.01365% | 0.01325% | 0.01873% |
| Nostoc sp. Lobaria pulmonaria (5183) cyanobiont | 0.00717% | 0.00570% | 0.00841% | 0.00669% | 0.00816% | 0.00772% | 0.01056% | 0.00779% | 0.00875% | 0.01759% | 0.01517% | 0.01902% | 0.02071% | 0.01363% | 0.01519% | 0.01397% | 0.01668% | 0.01124% | 0.01570% | 0.01951% | 0.01592% | 0.01852% | 0.01254% | 0.00885% | 0.01540% | 0.01522% |
| Calothrix sp. NIES-2098 | 0.00771% | 0.00984% | 0.01012% | 0.00952% | 0.01088% | 0.01185% | 0.01242% | 0.01407% | 0.01183% | 0.01985% | 0.01799% | 0.01832% | 0.02294% | 0.01682% | 0.01830% | 0.01894% | 0.01920% | 0.01223% | 0.01976% | 0.01440% | 0.01756% | 0.01875% | 0.01968% | 0.01025% | 0.01706% | 0.02010% |
| Calothrix sp. PCC 7507 | 0.01116% | 0.00570% | 0.01012% | 0.01003% | 0.01020% | 0.00931% | 0.01018% | 0.00929% | 0.00772% | 0.01511% | 0.01864% | 0.01815% | 0.02172% | 0.01213% | 0.02103% | 0.02127% | 0.02400% | 0.01091% | 0.01514% | 0.01345% | 0.02103% | 0.01578% | 0.01929% | 0.01158% | 0.01656% | 0.02010% |
| Leptolyngbya sp. NIES-3755 | 0.01222% | 0.01269% | 0.01228% | 0.01003% | 0.02879% | 0.00878% | 0.01279% | 0.00854% | 0.01080% | 0.02549% | 0.02080% | 0.01815% | 0.02071% | 0.01213% | 0.01460% | 0.01661% | 0.01417% | 0.01323% | 0.02105% | 0.01932% | 0.02062% | 0.01921% | 0.01177% | 0.00678% | 0.01524% | 0.01659% |
| Calothrix sp. NIES-4101 | 0.00717% | 0.01243% | 0.01137% | 0.01209% | 0.01587% | 0.01163% | 0.01254% | 0.01482% | 0.01029% | 0.02526% | 0.02622% | 0.01797% | 0.02396% | 0.01469% | 0.01908% | 0.02174% | 0.01714% | 0.01356% | 0.02400% | 0.02349% | 0.02245% | 0.01875% | 0.01929% | 0.00959% | 0.02302% | 0.02439% |
| Halothece sp. PCC 7418 | 0.00824% | 0.00699% | 0.00660% | 0.00592% | 0.01201% | 0.00571% | 0.00981% | 0.00779% | 0.00695% | 0.01782% | 0.01799% | 0.01797% | 0.01969% | 0.01512% | 0.01227% | 0.01428% | 0.01531% | 0.01521% | 0.02087% | 0.02159% | 0.02327% | 0.01304% | 0.01833% | 0.00976% | 0.01358% | 0.01776% |
| Stanieria sp. NIES-3757 | 0.00797% | 0.00648% | 0.00750% | 0.00746% | 0.02085% | 0.00709% | 0.01652% | 0.00703% | 0.00797% | 0.01353% | 0.02384% | 0.01745% | 0.02152% | 0.01363% | 0.02064% | 0.01848% | 0.01874% | 0.01124% | 0.01256% | 0.01572% | 0.01592% | 0.01715% | 0.01698% | 0.01034% | 0.01689% | 0.01873% |
| Nostocales cyanobacterium HT-58-2 | 0.01010% | 0.01010% | 0.00932% | 0.01106% | 0.01315% | 0.00963% | 0.00956% | 0.01256% | 0.01003% | 0.01511% | 0.01907% | 0.01727% | 0.02050% | 0.02491% | 0.01850% | 0.01382% | 0.01280% | 0.00694% | 0.01625% | 0.01345% | 0.01939% | 0.01738% | 0.01968% | 0.01414% | 0.01888% | 0.01698% |
| cyanobacterium endosymbiont of Epithemia turgida | 0.00292% | 0.00233% | 0.00432% | 0.00540% | 0.00929% | 0.00391% | 0.00447% | 0.00402% | 0.00386% | 0.01872% | 0.01538% | 0.01727% | 0.02822% | 0.01980% | 0.03349% | 0.02158% | 0.02034% | 0.00860% | 0.01920% | 0.01705% | 0.01980% | 0.02378% | 0.02469% | 0.01183% | 0.02468% | 0.01834% |
| *Oscillatoria nigro-viridis* | 0.01382% | 0.01321% | 0.01330% | 0.01106% | 0.01813% | 0.01153% | 0.01490% | 0.01105% | 0.01312% | 0.02481% | 0.02427% | 0.01692% | 0.02578% | 0.01512% | 0.01967% | 0.01568% | 0.01371% | 0.01852% | 0.02659% | 0.01932% | 0.02103% | 0.02058% | 0.01929% | 0.00868% | 0.01805% | 0.01541% |
| Cyanothece sp. ATCC 51142 | 0.00771% | 0.00751% | 0.00637% | 0.00592% | 0.01451% | 0.00529% | 0.01341% | 0.00553% | 0.00772% | 0.02414% | 0.01994% | 0.01640% | 0.02396% | 0.01639% | 0.01402% | 0.01692% | 0.01554% | 0.01190% | 0.02031% | 0.01743% | 0.01878% | 0.01944% | 0.01871% | 0.00926% | 0.01805% | 0.01951% |
| *Dolichospermum compactum* | 0.00505% | 0.00337% | 0.00398% | 0.00463% | 0.00861% | 0.00402% | 0.00447% | 0.00528% | 0.00334% | 0.01805% | 0.02535% | 0.01623% | 0.03593% | 0.00724% | 0.01051% | 0.01149% | 0.01234% | 0.01488% | 0.02050% | 0.02633% | 0.03552% | 0.02836% | 0.00579% | 0.00538% | 0.01126% | 0.01424% |
| *Calothrix parietina* | 0.00452% | 0.00933% | 0.00762% | 0.00874% | 0.01360% | 0.00783% | 0.00932% | 0.00754% | 0.00643% | 0.01692% | 0.01473% | 0.01605% | 0.02416% | 0.03598% | 0.03933% | 0.02205% | 0.02651% | 0.00794% | 0.01699% | 0.01818% | 0.01858% | 0.01990% | 0.02855% | 0.01059% | 0.02749% | 0.02829% |
| Nostoc sp. NIES-4103 | 0.01063% | 0.01166% | 0.01478% | 0.01440% | 0.01904% | 0.01121% | 0.01565% | 0.01407% | 0.01209% | 0.02707% | 0.02080% | 0.01570% | 0.02030% | 0.01554% | 0.01558% | 0.02407% | 0.01623% | 0.01521% | 0.01810% | 0.01837% | 0.02307% | 0.01418% | 0.02064% | 0.01017% | 0.01954% | 0.02107% |
| Fischerella sp. NIES-3754 | 0.00797% | 0.01295% | 0.00955% | 0.01029% | 0.01337% | 0.01047% | 0.01068% | 0.01005% | 0.00952% | 0.01263% | 0.01300% | 0.01535% | 0.01340% | 0.01299% | 0.01383% | 0.01149% | 0.01417% | 0.00761% | 0.01089% | 0.01023% | 0.01021% | 0.01349% | 0.01370% | 0.00695% | 0.01391% | 0.01444% |
| Synechococcus sp. PCC 7003 | 0.00478% | 0.00803% | 0.00387% | 0.00412% | 0.01247% | 0.00571% | 0.00360% | 0.00427% | 0.00720% | 0.02188% | 0.02124% | 0.01518% | 0.02294% | 0.01192% | 0.01538% | 0.01102% | 0.01463% | 0.01951% | 0.02105% | 0.01818% | 0.01715% | 0.01944% | 0.01312% | 0.00728% | 0.01308% | 0.01015% |
| *Pleurocapsa minor* | 0.00691% | 0.00622% | 0.00421% | 0.00797% | 0.03786% | 0.00508% | 0.00857% | 0.00703% | 0.00412% | 0.01399% | 0.01538% | 0.01361% | 0.01624% | 0.00873% | 0.01149% | 0.01118% | 0.00846% | 0.00761% | 0.01293% | 0.01099% | 0.01511% | 0.01692% | 0.01196% | 0.00554% | 0.01259% | 0.01522% |
| Anabaena sp. 54 | 0.00691% | 0.00414% | 0.00500% | 0.00566% | 0.01496% | 0.00423% | 0.00397% | 0.00452% | 0.00540% | 0.01353% | 0.02080% | 0.01361% | 0.02294% | 0.01661% | 0.01908% | 0.01304% | 0.01257% | 0.01157% | 0.01533% | 0.01913% | 0.02388% | 0.02241% | 0.01331% | 0.00959% | 0.01640% | 0.01502% |
| *Trichormus azollae* | 0.00478% | 0.00414% | 0.00364% | 0.00334% | 0.00703% | 0.00561% | 0.00596% | 0.00427% | 0.00566% | 0.01241% | 0.01430% | 0.01029% | 0.01766% | 0.00894% | 0.01207% | 0.01304% | 0.01326% | 0.00628% | 0.00960% | 0.01250% | 0.01266% | 0.01029% | 0.01022% | 0.00538% | 0.01524% | 0.01502% |
| *Cyanobacterium aponinum* | 0.00186% | 0.00181% | 0.08494% | 0.00103% | 0.00113% | 0.07552% | 0.09899% | 0.09897% | 0.00129% | 0.01060% | 0.00932% | 0.00872% | 0.00873% | 0.30593% | 0.00312% | 0.00342% | 0.00411% | 0.00099% | 0.01274% | 0.00909% | 0.01061% | 0.00686% | 0.00386% | 0.00074% | 0.29049% | 0.00429% |
| Nostoc sp. PCC 7107 | 0.00505% | 0.00440% | 0.00523% | 0.00334% | 0.00907% | 0.00391% | 0.00460% | 0.00528% | 0.00566% | 0.01128% | 0.01170% | 0.00872% | 0.01117% | 0.00979% | 0.00896% | 0.00838% | 0.00960% | 0.00331% | 0.00905% | 0.01383% | 0.00939% | 0.01075% | 0.00830% | 0.00554% | 0.00778% | 0.00663% |
| Prochlorococcus sp. MIT 0801 | 0.00053% | 0.00155% | 0.00193% | 0.00129% | 0.00159% | 0.00212% | 0.00211% | 0.00226% | 0.00334% | 0.00609% | 0.00715% | 0.00785% | 0.01035% | 0.01001% | 0.00682% | 0.00730% | 0.00846% | 0.00298% | 0.00868% | 0.00606% | 0.00653% | 0.00640% | 0.00984% | 0.00521% | 0.00679% | 0.00624% |
| *Anabaena cylindrica* | 0.07068% | 0.05984% | 0.05776% | 0.06404% | 0.17114% | 0.06346% | 0.07291% | 0.06732% | 0.06611% | 0.20414% | 0.20325% | 0.00663% | 0.00792% | 0.13753% | 0.14838% | 0.00404% | 0.15907% | 0.00231% | 0.00554% | 0.18259% | 0.00653% | 0.18981% | 0.16012% | 0.00132% | 0.15154% | 0.14986% |
| *Oscillatoria acuminata* | 0.12330% | 0.11243% | 0.00341% | 0.11574% | 0.00113% | 0.00360% | 0.00286% | 0.12108% | 0.11575% | 0.00564% | 0.00412% | 0.00611% | 0.00589% | 0.20651% | 0.18674% | 0.00357% | 0.19290% | 0.20137% | 0.00646% | 0.00720% | 0.00408% | 0.00412% | 0.00463% | 0.00157% | 0.19129% | 0.00449% |
| Synechococcus sp. PCC 73109 | 0.00159% | 0.00285% | 0.00182% | 0.00257% | 0.00227% | 0.00286% | 0.00186% | 0.00201% | 0.00386% | 0.00767% | 0.00607% | 0.00541% | 0.00731% | 0.00575% | 0.00253% | 0.00528% | 0.00549% | 0.00562% | 0.00369% | 0.00587% | 0.00572% | 0.00663% | 0.00637% | 0.00207% | 0.00530% | 0.00605% |
| Synechococcus sp. PCC 7002 | 0.00133% | 0.00130% | 0.00193% | 0.00283% | 0.00680% | 0.00148% | 0.00174% | 0.00126% | 0.00077% | 0.00451% | 0.00628% | 0.00541% | 0.00487% | 0.00192% | 0.00156% | 0.00217% | 0.00183% | 0.00661% | 0.00628% | 0.00606% | 0.00531% | 0.00435% | 0.00251% | 0.00141% | 0.00166% | 0.00215% |
| Nostoc sp. NIES-2111 | 0.00399% | 0.00492% | 0.00364% | 0.00257% | 0.00272% | 0.00286% | 0.00348% | 0.00352% | 0.00489% | 0.00383% | 0.00542% | 0.00454% | 0.00467% | 0.00404% | 0.00350% | 0.00450% | 0.00183% | 0.00364% | 0.00591% | 0.00777% | 0.00367% | 0.00320% | 0.00617% | 0.00240% | 0.00580% | 0.00527% |
| Anabaena sp. WA102 | 0.00452% | 0.00130% | 0.00557% | 0.00103% | 0.00703% | 0.00624% | 0.00882% | 0.00955% | 0.00154% | 0.02211% | 0.00325% | 0.00419% | 0.00487% | 0.01788% | 0.01869% | 0.00233% | 0.01531% | 0.02579% | 0.00499% | 0.00587% | 0.00408% | 0.00435% | 0.00347% | 0.00074% | 0.00149% | 0.01795% |
| Anabaena sp. 90 | 0.00133% | 0.00104% | 0.00671% | 0.00103% | 0.00136% | 0.00571% | 0.00621% | 0.00126% | 0.00129% | 0.01962% | 0.01994% | 0.00419% | 0.00751% | 0.01512% | 0.01752% | 0.00450% | 0.01257% | 0.01190% | 0.00683% | 0.00663% | 0.00592% | 0.00503% | 0.01640% | 0.00753% | 0.00050% | 0.00976% |
| *Arthrospira platensis* | 0.06537% | 0.05647% | 0.06459% | 0.00180% | 0.00181% | 0.05764% | 0.07440% | 0.06381% | 0.06276% | 0.22309% | 0.20412% | 0.00349% | 0.00487% | 0.14455% | 0.14546% | 0.00404% | 0.15336% | 0.00265% | 0.00351% | 0.00322% | 0.00510% | 0.20399% | 0.15221% | 0.00165% | 0.16247% | 0.16508% |
| Nostoc sp. PCC 7120 | - | - | - | - | - | 0.00011% | - | - | 0.00026% | 0.00023% | 0.00022% | 0.00052% | - | - | 0.00020% | 0.00031% | - | - | 0.00019% | - | - | 0.00023% | 0.00019% | - | 0.00017% | - |
| *Aulosira laxa* | 0.00027% | 0.00026% | - | - | - | - | - | - | - | - | 0.00022% | 0.00017% | - | 0.00043% | 0.00020% | - | 0.00023% | 0.00033% | - | 0.00038% | - | 0.00023% | 0.00019% | - | 0.00033% | 0.00020% |
| Calothrix sp. NIES-4071 | - | - | - | - | - | - | - | - | - | - | - | - | - | 0.00021% | - | - | - | - | - | - | - | - | - | - | - | - |
| Prochlorococcus sp. RS01 | - | - | - | - | - | - | - | 0.00025% | - | - | - | - | - | - | - | - | - | - | - | - | - | - | - | - | - | - |

Table S5 The BLAST result table.

| Query ID | Subject ID | Percentage of identical matches | Alignment length | Number of mismatches | Number of gap openings | Start of alignment in query | End of alignment in query | Start of alignment in subject | End of alignment in subject | Expected value | Bit score |
| --- | --- | --- | --- | --- | --- | --- | --- | --- | --- | --- | --- |
| lcl\|NZ_JTJD01000286.1_cds_WP_052456730.1_6136 | lcl\|EU140798.1_prot_ABX60154.1_3 | 27.2 | 81 | 49 | 2 | 1912 | 2124 | 307 | 387 | 4.18E-04 | 33.9 |
| lcl\|NZ_JTJD01000271.1_cds_WP_063776168.1_995 | lcl\|EU140798.1_prot_ABX60158.1_7 | 36.2 | 47 | 30 | 0 | 1591 | 1731 | 355 | 401 | 1.88E-05 | 37.7 |
| lcl\|NZ_JTJD01000271.1_cds_WP_063776168.1_995 | lcl\|EU140798.1_prot_ABX60154.1_3 | 34 | 47 | 31 | 0 | 1591 | 1731 | 357 | 403 | 3.28E-05 | 37 |
| lcl\|NZ_JTJD01000271.1_cds_WP_052288471.1_2619 | lcl\|EU140798.1_prot_ABX60161.1_10 | 25.7 | 105 | 72 | 3 | 283 | 582 | 2114 | 2217 | 1.11E-04 | 33.9 |
| lcl\|NZ_JTJD01000271.1_cds_WP_052288277.1_903 | lcl\|EU140798.1_prot_ABX60154.1_3 | 39.2 | 51 | 27 | 1 | 58 | 198 | 30 | 80 | 4.70E-05 | 35.8 |
| lcl\|NZ_JTJD01000271.1_cds_WP_044151064.1_743 | lcl\|EU140798.1_prot_ABX60161.1_10 | 27.3 | 183 | 111 | 4 | 931 | 1413 | 379 | 561 | 2.49E-07 | 43.9 |
| lcl\|NZ_JTJD01000271.1_cds_WP_039729963.1_2384 | lcl\|EU140798.1_prot_ABX60158.1_7 | 48.9 | 47 | 22 | 1 | 964 | 1104 | 350 | 394 | 3.53E-07 | 42.4 |
| lcl\|NZ_JTJD01000271.1_cds_WP_039729963.1_2384 | lcl\|EU140798.1_prot_ABX60154.1_3 | 51.4 | 37 | 18 | 0 | 964 | 1074 | 352 | 388 | 1.90E-06 | 40 |
| lcl\|NZ_JTJD01000271.1_cds_WP_039729315.1_299 | lcl\|EU140798.1_prot_ABX60154.1_3 | 20.7 | 429 | 310 | 10 | 40 | 1287 | 16 | 427 | 1.45E-12 | 59.7 |
| lcl\|NZ_JTJD01000271.1_cds_WP_039729315.1_299 | lcl\|EU140798.1_prot_ABX60158.1_7 | 19.4 | 427 | 312 | 10 | 40 | 1287 | 20 | 425 | 5.92E-12 | 57.8 |
| lcl\|NZ_JTJD01000271.1_cds_WP_039728691.1_3761 | lcl\|EU140798.1_prot_ABX60152.1_1 | 30.8 | 159 | 101 | 3 | 16 | 486 | 1463 | 1614 | 4.17E-12 | 56.6 |
| lcl\|NZ_JTJD01000271.1_cds_WP_039728691.1_3761 | lcl\|EU140798.1_prot_ABX60163.1_12 | 31 | 158 | 102 | 3 | 19 | 486 | 1253 | 1405 | 1.07E-09 | 49.3 |
| lcl\|NZ_JTJD01000271.1_cds_WP_039728691.1_3761 | lcl\|EU140798.1_prot_ABX60162.1_11 | 32.9 | 155 | 96 | 4 | 31 | 486 | 1484 | 1633 | 2.93E-13 | 60.1 |
| lcl\|NZ_JTJD01000271.1_cds_WP_039728387.1_3435 | lcl\|EU140798.1_prot_ABX60161.1_10 | 23.2 | 555 | 374 | 23 | 289 | 1881 | 35 | 561 | 1.27E-16 | 74.7 |
| lcl\|NZ_JTJD01000271.1_cds_WP_039726789.1_1968 | lcl\|EU140798.1_prot_ABX60163.1_12 | 30 | 297 | 183 | 5 | 7 | 825 | 565 | 860 | 1.39E-30 | 112 |
| lcl\|NZ_JTJD01000271.1_cds_WP_039726789.1_1968 | lcl\|EU140798.1_prot_ABX60162.1_11 | 32.5 | 289 | 176 | 8 | 7 | 822 | 545 | 831 | 2.41E-33 | 120 |
| lcl\|NZ_JTJD01000271.1_cds_WP_039726789.1_1968 | lcl\|EU140798.1_prot_ABX60153.1_2 | 33.9 | 286 | 175 | 6 | 7 | 825 | 550 | 834 | 7.72E-33 | 119 |
| lcl\|NZ_JTJD01000271.1_cds_WP_039726789.1_1968 | lcl\|EU140798.1_prot_ABX60161.1_10 | 32.2 | 286 | 176 | 6 | 13 | 816 | 1209 | 1494 | 3.26E-31 | 114 |
| lcl\|NZ_JTJD01000271.1_cds_WP_039726789.1_1968 | lcl\|EU140798.1_prot_ABX60152.1_1 | 32.6 | 279 | 179 | 4 | 13 | 825 | 589 | 866 | 5.46E-36 | 128 |
| lcl\|NZ_JTJD01000271.1_cds_WP_039726338.1_1437 | lcl\|EU140798.1_prot_ABX60161.1_10 | 23 | 187 | 120 | 3 | 178 | 678 | 49 | 231 | 1.88E-06 | 41.6 |
| lcl\|NZ_JTJD01000271.1_cds_WP_039726150.1_1207 | lcl\|EU140798.1_prot_ABX60164.1_13 | 52.4 | 143 | 67 | 1 | 7 | 432 | 24 | 166 | 1.94E-46 | 143 |
| lcl\|NZ_JTJD01000271.1_cds_WP_039725574.1_507 | lcl\|EU140798.1_prot_ABX60163.1_12 | 29.5 | 268 | 170 | 5 | 364 | 1113 | 194 | 460 | 1.99E-24 | 96.3 |
| lcl\|NZ_JTJD01000271.1_cds_WP_039725574.1_507 | lcl\|EU140798.1_prot_ABX60162.1_11 | 28.3 | 265 | 172 | 6 | 364 | 1113 | 187 | 448 | 9.27E-23 | 91.3 |
| lcl\|NZ_JTJD01000271.1_cds_WP_039725553.1_480 | lcl\|EU140798.1_prot_ABX60152.1_1 | 24.5 | 440 | 276 | 10 | 25 | 1242 | 65 | 482 | 8.92E-26 | 101 |
| lcl\|NZ_JTJD01000271.1_cds_WP_039725553.1_480 | lcl\|EU140798.1_prot_ABX60153.1_2 | 25.8 | 434 | 282 | 9 | 13 | 1239 | 34 | 452 | 1.67E-37 | 136 |
| lcl\|NZ_JTJD01000271.1_cds_WP_039725553.1_480 | lcl\|EU140798.1_prot_ABX60163.1_12 | 26.6 | 433 | 266 | 11 | 43 | 1242 | 47 | 460 | 1.96E-32 | 121 |
| lcl\|NZ_JTJD01000271.1_cds_WP_039725553.1_480 | lcl\|EU140798.1_prot_ABX60162.1_11 | 25.6 | 429 | 279 | 10 | 25 | 1242 | 37 | 448 | 1.87E-33 | 124 |
| lcl\|NZ_JTJD01000271.1_cds_WP_039725553.1_480 | lcl\|EU140798.1_prot_ABX60161.1_10 | 27.3 | 278 | 172 | 7 | 469 | 1242 | 842 | 1109 | 2.37E-19 | 81.6 |
| lcl\|NZ_JTJD01000271.1_cds_WP_039725510.1_419 | lcl\|EU140798.1_prot_ABX60161.1_10 | 28.1 | 139 | 87 | 5 | 241 | 627 | 2085 | 2220 | 5.46E-08 | 44.7 |
| lcl\|NZ_JTJD01000271.1_cds_WP_039725447.1_335 | lcl\|EU140798.1_prot_ABX60156.1_5 | 43.2 | 431 | 237 | 3 | 10 | 1290 | 11 | 437 | 4.60E-112 | 330 |
| lcl\|NZ_JTJD01000264.1_cds_WP_044150597.1_6664 | lcl\|EU140798.1_prot_ABX60165.1_14 | 24.7 | 393 | 253 | 13 | 4 | 1086 | 3 | 384 | 5.64E-20 | 82 |
| lcl\|NZ_JTJD01000259.1_cds_WP_052456634.1_7254 | lcl\|EU140798.1_prot_ABX60158.1_7 | 28.9 | 128 | 74 | 5 | 571 | 930 | 270 | 388 | 1.49E-06 | 40 |
| lcl\|NZ_JTJD01000259.1_cds_WP_052456634.1_7254 | lcl\|EU140798.1_prot_ABX60154.1_3 | 29.1 | 127 | 81 | 3 | 571 | 930 | 266 | 390 | 2.26E-10 | 52 |
| lcl\|NZ_JTJD01000258.1_cds_WP_039724761.1_4357 | lcl\|EU140798.1_prot_ABX60165.1_14 | 23 | 257 | 151 | 10 | 1804 | 2484 | 16 | 255 | 2.19E-04 | 35.8 |
| lcl\|NZ_JTJD01000258.1_cds_WP_039724654.1_4494 | lcl\|EU140798.1_prot_ABX60161.1_10 | 36.2 | 127 | 67 | 4 | 145 | 498 | 2103 | 2224 | 3.27E-11 | 55.8 |
| lcl\|NZ_JTJD01000238.1_cds_WP_044149788.1_5647 | lcl\|EU140798.1_prot_ABX60162.1_11 | 22.1 | 190 | 102 | 8 | 37 | 525 | 1484 | 1654 | 7.63E-04 | 31.2 |
| lcl\|NZ_JTJD01000238.1_cds_WP_044149788.1_5647 | lcl\|EU140798.1_prot_ABX60152.1_1 | 32 | 50 | 34 | 0 | 13 | 162 | 1460 | 1509 | 1.41E-04 | 33.5 |
| lcl\|NZ_JTJD01000237.1_cds_WP_052456611.1_4744 | lcl\|EU140798.1_prot_ABX60164.1_13 | 52.9 | 68 | 32 | 0 | 31 | 234 | 124 | 191 | 9.54E-24 | 81.3 |
| lcl\|NZ_JTJD01000217.1_cds_WP_044149254.1_5117 | lcl\|EU140798.1_prot_ABX60157.1_6 | 23.9 | 134 | 94 | 4 | 13 | 405 | 1 | 129 | 4.61E-05 | 33.1 |
| lcl\|NZ_JTJD01000204.1_cds_WP_039722426.1_4953 | lcl\|EU140798.1_prot_ABX60161.1_10 | 26.9 | 134 | 86 | 3 | 22 | 387 | 2082 | 2215 | 2.93E-06 | 38.9 |
| lcl\|NZ_JTJD01000201.1_cds_WP_044148789.1_8568 | lcl\|EU140798.1_prot_ABX60161.1_10 | 27.1 | 543 | 339 | 9 | 10 | 1485 | 25 | 561 | 3.75E-52 | 182 |
| lcl\|NZ_JTJD01000167.1_cds_WP_044148321.1_8529 | lcl\|EU140798.1_prot_ABX60161.1_10 | 27 | 222 | 108 | 11 | 822 | 301 | 241 | 456 | 3.62E-04 | 33.9 |
| lcl\|NZ_JTJD01000101.1_cds_WP_052456421.1_5034 | lcl\|EU140798.1_prot_ABX60165.1_14 | 23.6 | 343 | 248 | 9 | 1 | 1026 | 18 | 347 | 1.82E-20 | 84.3 |
| lcl\|NZ_JTJD01000089.1_cds_WP_044146470.1_7321 | lcl\|EU140798.1_prot_ABX60161.1_10 | 31 | 113 | 62 | 4 | 124 | 432 | 2111 | 2217 | 1.87E-04 | 33.1 |
| lcl\|NZ_JTJD01000081.1_cds_WP_044146359.1_7205 | lcl\|EU140798.1_prot_ABX60152.1_1 | 29 | 379 | 243 | 8 | 7 | 1128 | 85 | 442 | 2.07E-31 | 118 |
| lcl\|NZ_JTJD01000081.1_cds_WP_044146359.1_7205 | lcl\|EU140798.1_prot_ABX60163.1_12 | 29 | 376 | 245 | 7 | 142 | 1236 | 96 | 460 | 3.18E-33 | 123 |
| lcl\|NZ_JTJD01000081.1_cds_WP_044146359.1_7205 | lcl\|EU140798.1_prot_ABX60153.1_2 | 29.7 | 370 | 239 | 7 | 142 | 1233 | 98 | 452 | 8.46E-35 | 128 |
| lcl\|NZ_JTJD01000081.1_cds_WP_044146359.1_7205 | lcl\|EU140798.1_prot_ABX60161.1_10 | 28 | 282 | 181 | 6 | 436 | 1236 | 835 | 1109 | 9.54E-25 | 98.2 |
| lcl\|NZ_JTJD01000081.1_cds_WP_044146359.1_7205 | lcl\|EU140798.1_prot_ABX60162.1_11 | 33.1 | 275 | 175 | 4 | 424 | 1236 | 179 | 448 | 5.92E-33 | 122 |
| lcl\|NZ_JTJD01000053.1_cds_WP_044145794.1_6294 | lcl\|EU140798.1_prot_ABX60158.1_7 | 32.2 | 87 | 52 | 3 | 40 | 288 | 302 | 385 | 6.94E-05 | 32.3 |
| lcl\|NZ_JTJD01000022.1_cds_WP_044145275.1_7796 | lcl\|EU140798.1_prot_ABX60162.1_11 | 32 | 122 | 64 | 5 | 28 | 354 | 1482 | 1597 | 2.27E-05 | 36.2 |
| lcl\|NZ_JTJD01000022.1_cds_WP_044145275.1_7796 | lcl\|EU140798.1_prot_ABX60152.1_1 | 27.6 | 105 | 58 | 4 | 25 | 306 | 1465 | 1562 | 8.63E-04 | 31.2 |


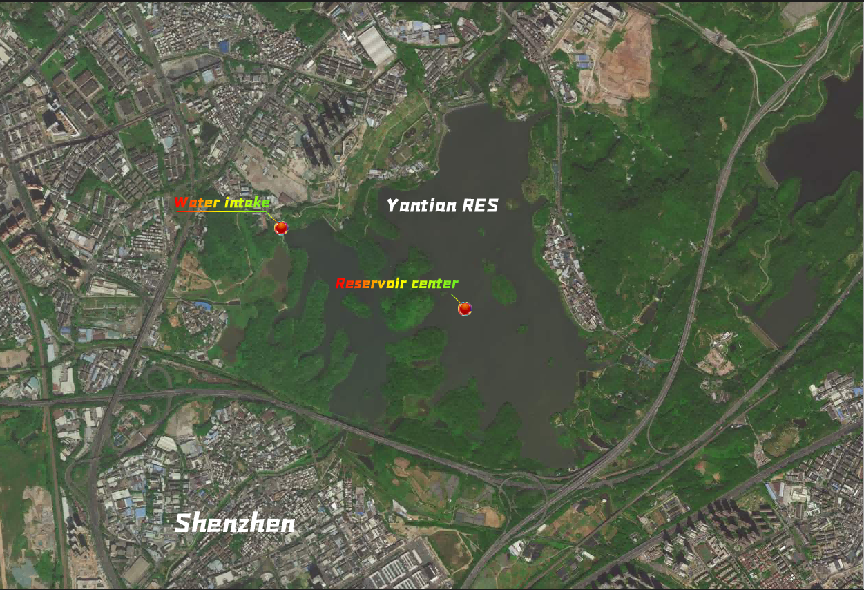


Figure S1


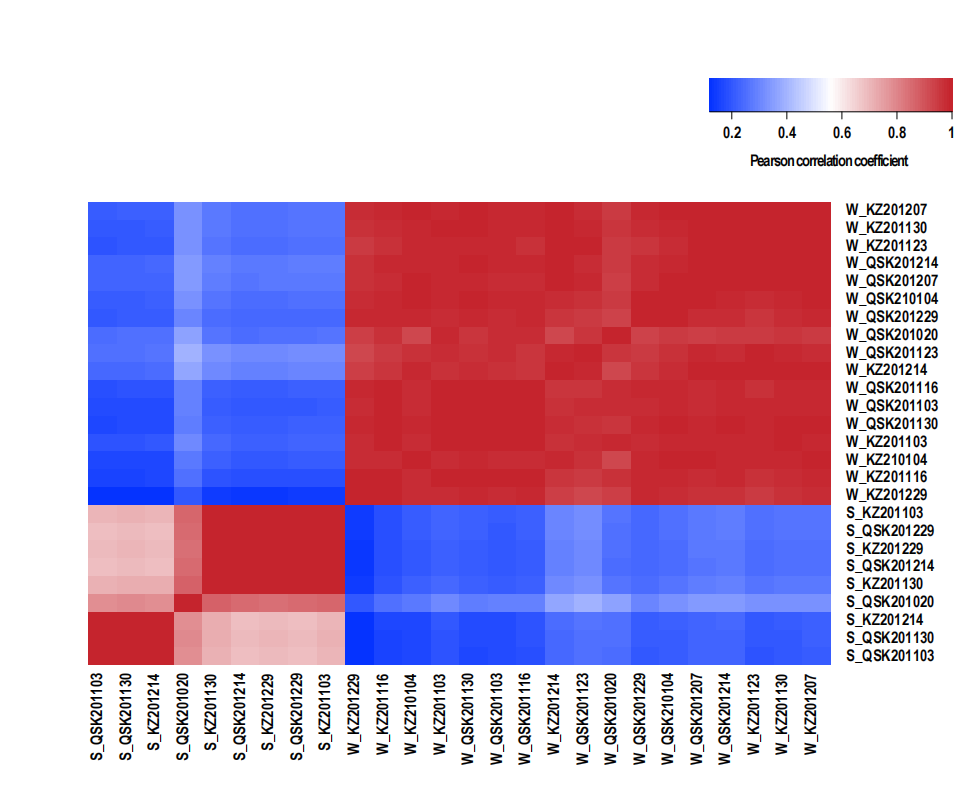


Figure S2


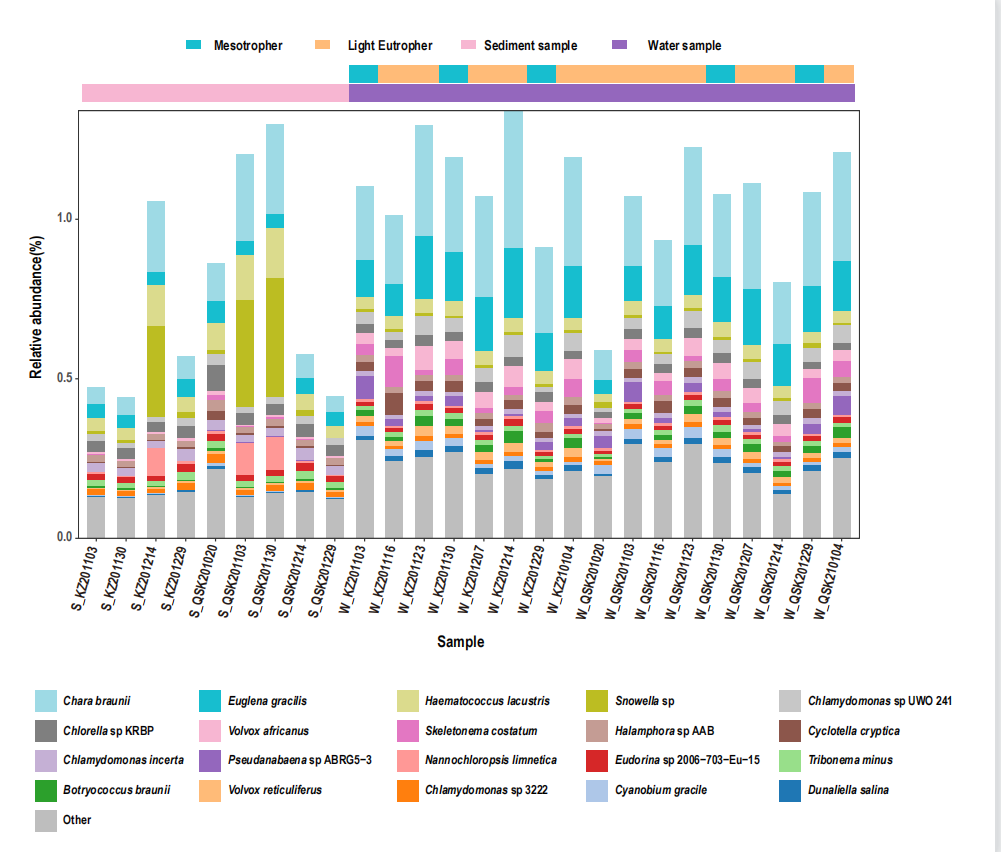


Figure S3

Figure S4


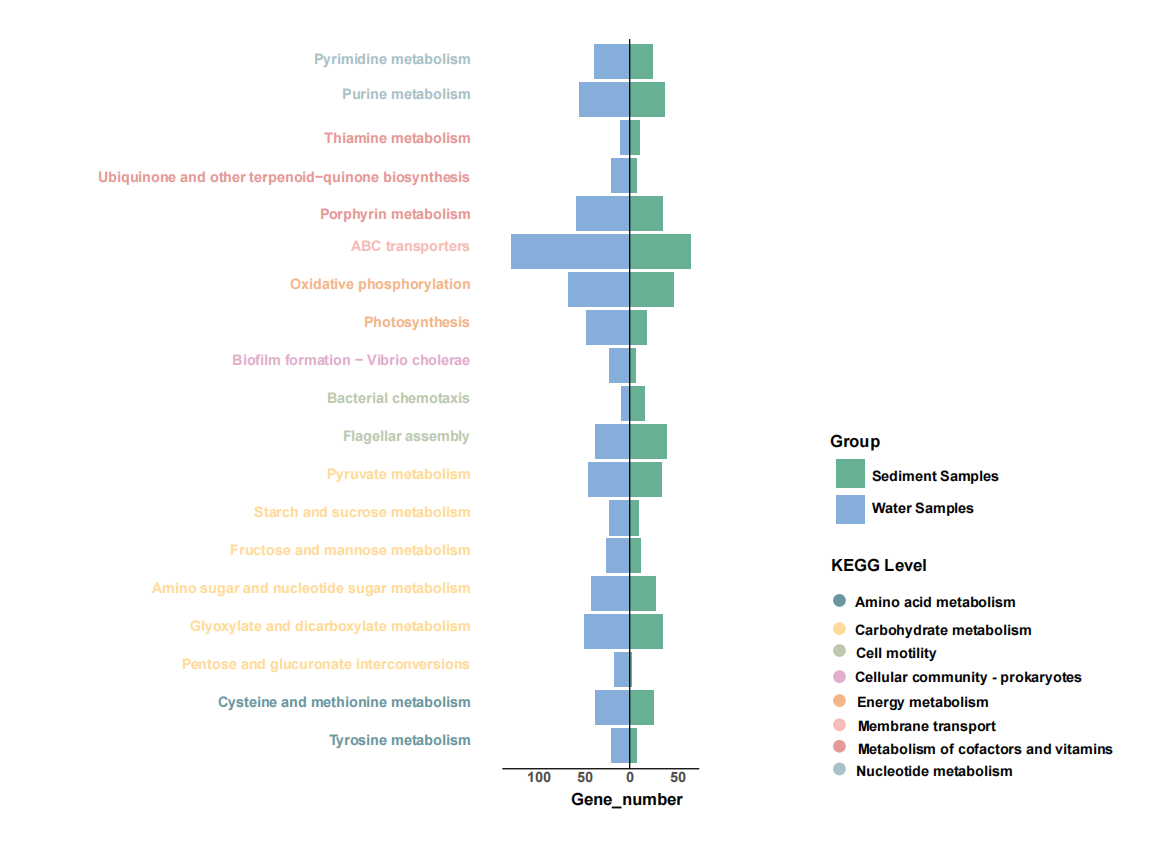


Figure S5


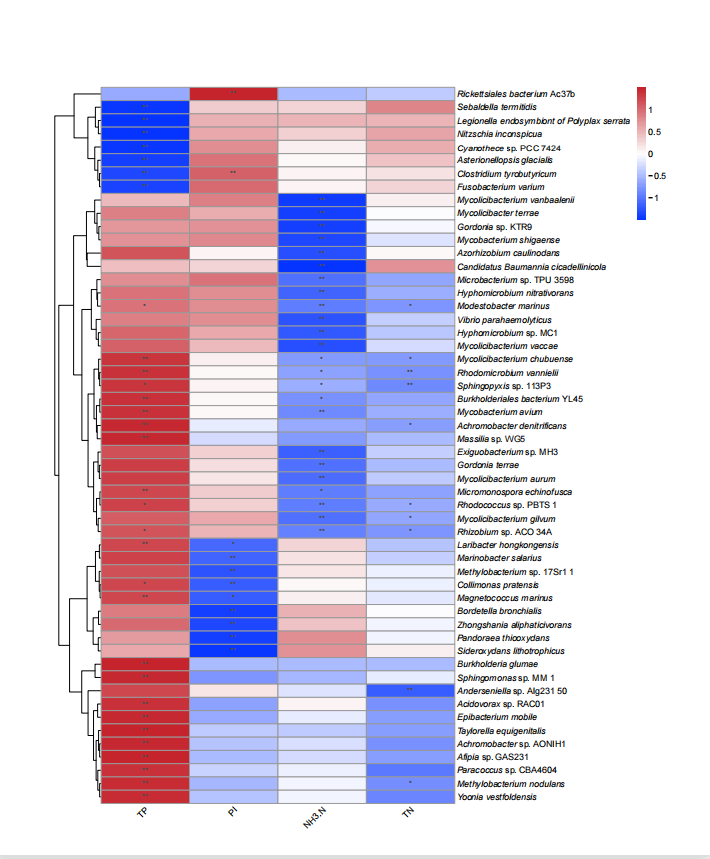


Figure S6


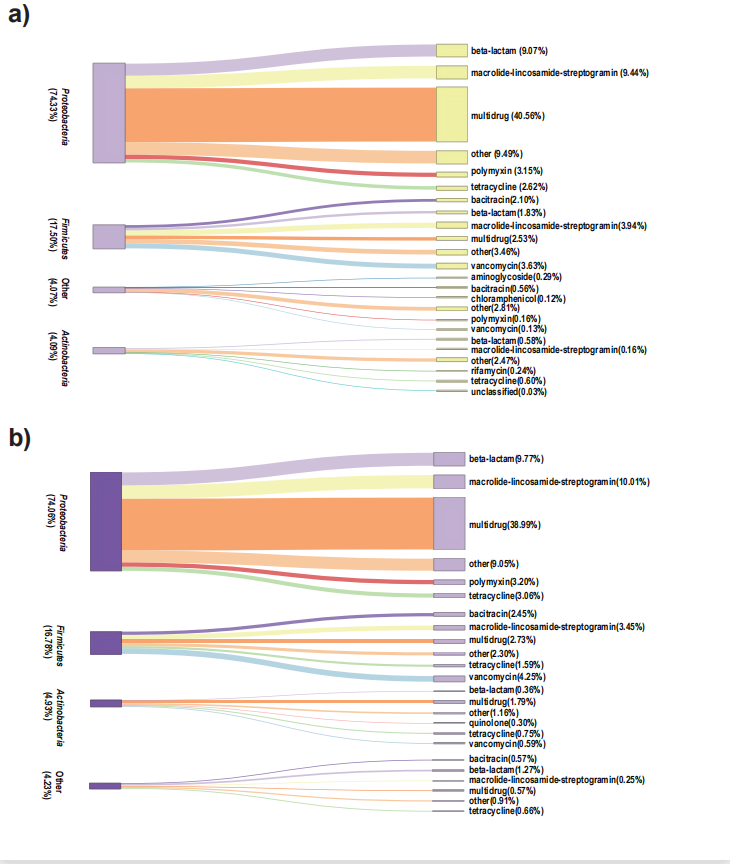


Figure S7


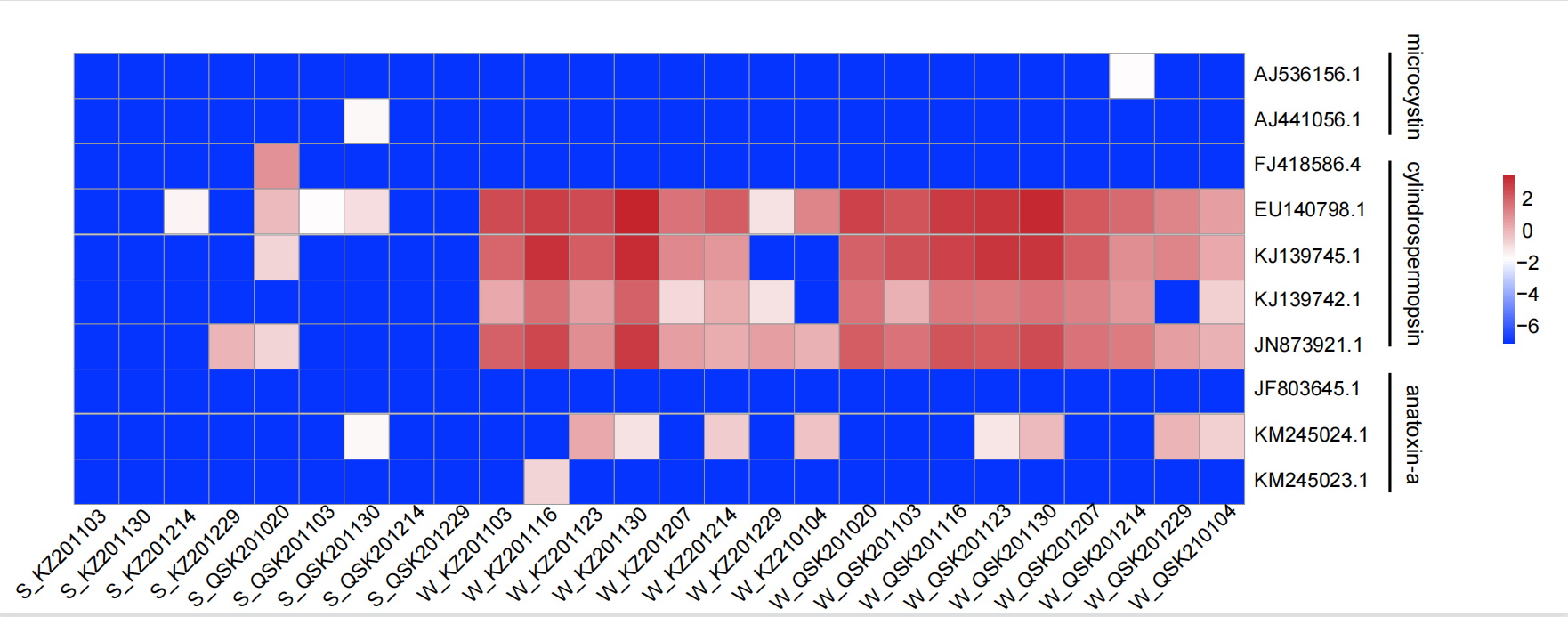


Figure S8
